# Nonequilibrium Binding Free Energy Simulations: Minimizing Dissipation

**DOI:** 10.1021/acs.jctc.4c01453

**Published:** 2025-02-05

**Authors:** Eleonora Serra, Alessia Ghidini, Sergio Decherchi, Andrea Cavalli

**Affiliations:** † Department of Pharmacy and Biotechnology (FaBiT), Alma Mater Studiorum-University of Bologna, via Belmeloro 6, 40126 Bologna, Italy; ‡ Computational & Chemical Biology, Fondazione Istituto Italiano di Tecnologia, via Morego 30, 16163 Genoa, Italy; § Centre Européen de Calcul Atomique et Moléculaire (CECAM), Ecole Polytechnique Fédérale de Lausanne, 1015 Lausanne, Switzerland; ∥ Data Science and Computation Facility, Fondazione Istituto Italiano di Tecnologia, via Morego 30, 16163 Genoa, Italy

## Abstract

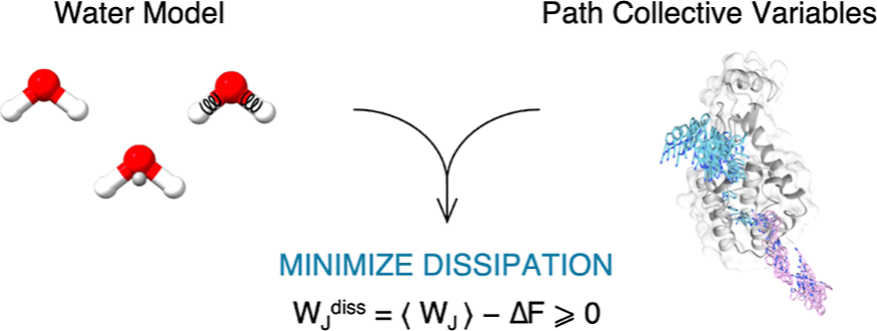

While nowadays approaches for equilibrium free energy estimation are well established, nonequilibrium simulations represent both an appealing computational opportunity and a challenge. This kind of simulations allows for a trivially parallel scheme, but at the same time the significant amount of irreversible work often generated during the steering process (either alchemical or physical) can hinder the convergence of free energy estimators. Here, we discuss in depth this issue for the protein–ligand binding free energy estimation carried out via physical paths. We found that the water model and the parametrization of the path collective variables have a remarkable impact on the convergence rate of the estimators (e.g., Crooks). Finally, we provide practical recipes to enhance the convergence speed and minimize dissipation.

## 1 Introduction

In the realm of biochemistry and biophysics, the free energy difference between two equilibrium states (*A* and *B*) of a system, Δ*F*_*AB*_ = *F*(*B*) – *F*(*A*), is among the most pivotal thermodynamic quantities. To estimate Δ*F*_*AB*_ using computer simulations, numerous methods have been developed. Initially, these methods relied on equilibrium sampling as in the case of thermodynamic integration^1^ and free energy perturbation.^2^ In the last decades, increasing attention has also been given to nonequilibrium methods.^3^

During a nonequilibrium simulation, an equilibrated system is externally driven away from equilibrium along a specific collective variable (ξ), generating an amount of work that will depend on the initial conditions of the simulations and the followed transformation protocol. In this process, a useful quantity to be measured is the Jarzynski work, *W*_J_.^4,5^ For a system that evolves along the collective variable ξ, *W*_J_ corresponds to the path integral of  along the trajectory Γ_t_
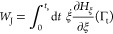
1where *H*_ξ_ is the modified Hamiltonian of the system, including the time-varying driving potential added to the regular potential. During irreversible simulations, *W*_J_ accounts for the cumulative change of the Hamiltonian of the system in time only.^4^ Considering the second law of thermodynamics, for a quasi-static ideal transformation this work amount corresponds to the free energy difference Δ*F*_*AB*_, while for nonequilibrium irreversible, real-world, transformations the work will exceed on average the free energy difference by a quantity defined as the dissipated work (*W*_J_^diss^)^5^
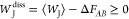
2

The Jarzynski work encountered during a single simulation is a random variable. Thus, it can be decomposed into its deterministic part, namely the free energy difference, plus the incurred dissipation (*W*_J_^diss,traj^), leading to
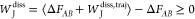
3

To calculate Δ*F*_*AB*_ from nonequilibrium simulations, estimators need to consider the *W*_J_ resulting from several nonequilibrium trajectories. The first nonequilibrium estimator developed is the Jarzynski equality,^5,6^ which relates the equilibrium free energy difference between the initial and final states of the system to the external *W*_J_ via an exponential average relation
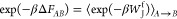
4Here, β = 1/*k*_B_*T*, *k*_B_ is the Boltzmann constant, and *T* is the absolute temperature of the simulated system. The angular brackets specify an exponential average over trajectories where the system evolves from its initial state *A* to its target state *B* with the same protocol (same variation of parameters over time along the same path from *A* to *B*). Therefore, the Jarzynski equality is defined as a unidirectional estimator. The exponential average is estimated via *N* sampled trajectories.

Although the Jarzynski equality has been widely used for free energy estimates, it has been proven to have statistical convergence difficulties.^7^ This happens because the Jarzynski equality consists of an exponential average, dominated by low *W*_J_ values that are rarely sampled. Consequently, the number of finite-time simulations required to achieve reasonable convergence might become prohibitively large.

To improve the accuracy of estimates, the Crooks Fluctuation Theorem (CFT)^8,9^ can be employed, combining data from forward and backward transformations. This bidirectional nonequilibrium estimator is defined as
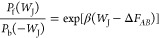
5where *P*_f_(*W*_J_) and *P*_b_(−*W*_J_) are respectively the forward *W*_J_ and backward *–W*_J_ distributions, i.e., the distribution of the Jarzynski work obtained from *A* to *B* and vice versa. The graphical interpretation of this equation is that the estimated free energy lies at the intersection point of *P*_f_(*W*_J_) and *P*_b_(−*W*_J_). Similarly to the Jarzynski equality, the CFT is negatively affected by dissipation. Nevertheless, the CFT achieves higher efficiency due to its bidirectional nature. To accurately estimate Δ*F*_*AB*_ using the CFT, it is sufficient to reliably determine the intersection point of the forward and backward distributions, whereas in the Jarzynski case, one needs to accurately sample the tail of the forward (or backward) distribution. Hence, the CFT bidirectionality allows the dissipated work to be more easily discounted by the estimation process. As emphasized by Pohorille et al.,^10^ albeit bidirectional, given a fixed amount of available computational time, the CFT proves to be more reliable in producing free energy difference estimates than the Jarzynski estimator.

Nonequilibrium estimators have been widely used in free energy calculations following either alchemical routes^11,12^ or path-based strategies.^13^ A notable example is the work by de Groot’s group on nonequilibrium alchemical transformations. In their approach, equilibrium simulations are conducted only for the two physical end states, while nonequilibrium transitions are used to connect them through alchemical space. Then, the Jarzynski equality or the CFT is used to recover the free energy differences between the end states.^14^

However, the practical applicability of these nonequilibrium estimators is often limited by convergence issues. In particular, their ability to efficiently converge to the correct estimate critically depends on the amount of incurred dissipation during simulations. This is evident by replacing in the Jarzynski equality (eq 4) the *W*_J_ definition from eq 2
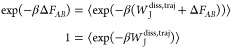
6where a zero dissipation would lead to a straightforward identity.

Given an ensemble of irreversible transformations, the lower the dissipated work, the lower the *W*_J_ associated with the process; hence, the faster the convergence of nonequilibrium estimators to the true free energy difference. Reducing the dissipated work during finite-time transformations becomes pivotal to improving convergence and obtaining efficient nonequilibrium strategies. Moreover, the dissipated work not only inherently depends on the transformation route but also on the physicochemical parameters of the system of interest (eventually the end points Hamiltonians). Hence, these are key factors to be taken into account when devising nonequilibrium protocols.

Nonequilibrium strategies employing work values are computationally appealing due to their inherently trivially parallelizable nature. Thus, they are rather tempting for computationally demanding simulations like the ones involving protein–ligand binding studies, i.e., estimating binding free energies, Δ*F*_b_. Δ*F*_b_ is the free energy difference related to the binding process of a drug to a target and directly correlates with the drug’s biological affinity for the protein, representing a critical quantity for biochemistry and drug discovery.^15^ Although equilibrium strategies are commonly applied, recent interest has grown in the application of nonequilibrium methods, particularly following an alchemical or physical observable-based route performing Steered Molecular Dynamics (SMD) simulations.^11,12,16,17^

In our recent work, we investigated the potentiality of coupling nonequilibrium SMD methods and Path Collective Variables (PCVs).^18^ Leveraging new parallel and GPU-based architectures, this strategy proved feasible with straightforward parallelization of simulations and accuracy in estimating Δ*F*_b_. In this previous work, we demonstrated that the CFT estimator exhibits greater robustness and is less affected by convergence issues compared to the Jarzynski one. Moreover, as expected, we confirmed that slower protocols, characterized by lower pulling speeds, are associated with a higher probability of sampling the tail of the Jarzynski work distribution, allowing convergence to be more easily achieved.

According to ref (10), a rough estimate of the number of samples required for convergence of nonequilibrium estimators is closely related to the amount of dissipated work during transformations via an exponential relation. Consequently, as the dissipation increases, the number of simulations needed to achieve convergence of Δ*F*_*AB*_ rapidly becomes unfeasible. Evidently, the applicability of our nonequilibrium protocol is directly linked to the capability of minimizing the dissipated work during the process.

Here, we investigate two important factors contributing to the dissipation encountered during irreversible SMD simulations, namely the choice of the water model and the PCVs definition. We provide suggestions of general applicability in nonequilibrium simulations, particularly regarding the water model choice, whereas for PCVs we present considerations specific to our protocol. Overall, the presented recipes significantly improve the convergence and accuracy of the nonequilibrium estimation process.

### 1.1 Water Models

In protein–ligand binding simulations, the aqueous environment contributes significantly to the potential and kinetic energy of the system, as it usually represents more than 90% of the solvated system. Therefore, a correct description of water behavior is crucial for achieving quantitative results from Molecular Dynamics (MD) simulations.^19,20^ Fully atomistic explicit water models, despite being more computationally demanding than implicit ones, provide a higher level of detail and accuracy, required for binding simulations.^15^ However, no explicit water model can perfectly replicate all experimental properties of water simultaneously.^21^ Thus, the selection of an appropriate solvent model requires careful consideration of its ability to reproduce the specific characteristics of water relevant to the studied phenomena.^22,23^

An explicit water model is defined as the force field (FF) potential energy function and a set of force field parameters to describe intra- and intermolecular interactions of water clusters and aqueous environments. The most employed category of water models is the fixed-charge *N*-point water models, where *N* is an integer ranging from 3 to 6 indicating the number of interaction sites. These models use the Lennard-Jones (LJ) 12-6 potential for dispersion and repulsion interactions, while the Coulombic potential for pairwise interactions between intermolecular pairs of point charges. The general pairwise potential energy function for these water models is defined as follows
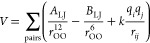
7Here, *r*_OO_ represents the oxygen–oxygen distances, *A*_LJ_ and *B*_LJ_ are the LJ parameters, *q*_*i*_ and *q*_*j*_ are the charges of the *i*th and *j*th charged sites at distance *r*_*ij*_, and *k* is the Coulomb’s electrostatic constant. The geometrical and pairwise energy function parameters of water molecules are determined through a fitting procedure against specific experimental properties of water (often at the expense of others). Early water models employ a rigid geometry closely resembling actual water molecules, where the OH bond length is 0.9572 Å and the HOH angle is 104.52°. Starting from rigid models, flexible variants have been designed to accommodate fluctuations in the geometry of water molecules (i.e., bond stretching and angle bending) through harmonic potentials.

Over the years, various water models have been developed, each with parameters tuned to replicate experimental properties of water, including but not limited to density, the self-diffusion coefficient, and the dielectric constant. However, the parameters of a specific water model are often suboptimal in reproducing jointly all experimental properties of water, leading to inaccuracies in MD simulation results.^24^

Despite more advanced and polarizable water models have been developed, the most commonly used in protein–ligand binding simulations are the fixed-charge 3- and 4-point models, which offer a trade-off between accuracy and computational costs.

#### 1.1.1 3-Point Water Models

3-point water models represent water monomers with one oxygen and two hydrogen atoms, each assigned a fixed atomic partial charge placed on the nuclei. Although these are the simplest models, they are among the most widely used because of their optimal compromise between accuracy and speed: they have an accuracy limit in the reproduction of some bulk water properties but allow for computational efficiency. 3-Point models are characterized by 6 independent parameters: the OH distance, the HOH angle, the hydrogen and oxygen point charges, and the two LJ parameters. The LJ parameter is placed on the oxygen atom, while van der Waals interactions among hydrogen atoms are not parametrized. Following diverse parametrization strategies, different 3-point models were developed. Force field parameters for 3-point water models discussed here are reported in Table 1.

**Table 1:** Force Field Parameters for 3-Point Water Models^a^

model	O σ (Å)	O ϵ (kcal/mol)	O charge (e)	H charge (e)	OH bond (Å)	H–O–H angle (deg)
TIP3P	3.15061	0.1521	–0.834	0.417	0.9572	104.52
TIP3P/Fw	3.15061	0.1521	–0.834	0.417	0.9572	104.52
OPC3	3.17427	0.163406	–0.89517	0.447585	0.97888	109.47

a For the flexible model, the additional parameters are *k*_b_ = 529.581 kcal mol^–1^ Å^–2^ and *k*_θ_ = 50.0 kcal mol^–1^ rad^–2^.

The Transferable Intermolecular Potential 3-Point (TIP3P) model is one of the first explicit solvent models developed for biomolecular simulations and is based on the experimental geometry of water monomers in gas phase.^25^ This rigid nonpolarizable model reproduces well some key features of bulk water, such as enthalpy of vaporisation and dielectric constant at 298 K and 1 bar. However, it underestimates the density and overestimates the self-diffusion coefficient. TIP3P has historically been one of the most popular choices for simulating proteins in solution, primarily due to its simplicity and effectiveness. For this reason, our systematic study of water models initially considers simulations in a TIP3P environment.

A flexible version of the TIP3P force field (TIP3P/Fw) was developed to incorporate the flexibility of water by introducing intramolecular potential parameters.^26^ This enhancement aims to improve the description of water interactions; however, flexible models come with a reduction in computational efficiency compared to their rigid counterparts. In particular, the introduction of flexibility requires a reduction in the integration time step from 2 fs (possible with rigid models by constraining chemical bonds involving hydrogen atoms) to 1 fs. Although doubling the computational cost, this does not always lead to better accuracy in reproducing water features in comparison to rigid models.

A relatively recent development in water modeling is represented by the 3-point model Optimal Point Charge (OPC3).^27^ OPC3 diverges from the TIP3P model in the pursuit of optimal parameters: instead of relying on traditional geometric constraints or predefined point charge values/positions, OPC3 focuses on achieving accuracy in reproducing the electrostatic field resulting from the correct charge distribution. OPC3 was indeed parametrized based on the principle that accurately reproducing electrostatic interactions is essential for capturing hydrogen bonding and other characteristics of liquid water. In ref (24) OPC3 is shown to surpass commonly used water models in its class by delivering significantly improved accuracy across a wide range of temperatures. By completely abandoning constraints on point charge geometry, OPC3 marks the achievement of the accuracy limit for nonpolarizable 3-point models concerning bulk water properties.^27^

#### 1.1.2 4-Point Water Models

4-point models represent water monomers by one oxygen atom, two hydrogen atoms, and one dummy atom. Differently to 3-point models, the negative point charge is not centered on the oxygen atom but shifted along the HOH bisector toward the hydrogen atoms by 0.15 Å, denoted by a mass-less dummy atom near the oxygen atom. This accounts for the offset of the partial charge on the oxygen atom and better mimics the quadrupole moment of the water molecule, improving the representation of the electrostatic potential. 4-Point models are characterized by 7 independent parameters: the OH distance, the HOH angle, the distance between the oxygen and the dummy atom, the point charges of the hydrogen and the dummy atom, and the two LJ parameters. Different 4-point models were obtained following different parametrization strategies, including TIP4P, its flexible version TIP4P/Fw, and OPC. Force field parameters for these 4-point water models are reported in Table 2.

**Table 2:** Force Field Parameters for 4-Point Water Models^a^

model	O σ (Å)	O ϵ (kcal/mol)	O charge (e)	H charge (e)	OH bond (Å)	H–O–H angle (deg)	OM bond (Å)
TIP4P	3.1565	0.155	–1.04	0.52	0.9572	104.52	0.15
TIP4P/Fw	3.1565	0.155	–1.04	0.52	0.9572	104.52	0.15
OPC	3.16655	0.212801	–1.3582	0.6791	0.8724	103.6	0.1594

a For the flexible model, the additional parameters are *k*_b_ = 103.389 kcal mol^–1^ Å^–2^ and *k*_θ_ = 2.287 kcal mol^–1^ rad^–2^.

The Transferable Intermolecular Potential 4-Point (TIP4P) model represents a modification of the original TIP3P model, adopting the same 3-charge rigid planar structure but introducing a dummy atom bearing the negative point charge.^28^ While this modification improves the geometry of water dimers, it results in an underestimation of the dielectric constant.

A flexible variant of the TIP4P model (TIP4P/Fw) was also developed, introducing flexibility with a Morse potential for bond stretching and a harmonic term for angle bending.^29^ Furthermore, a slight adjustment has been made to the parameters of the LJ potential for intermolecular interactions. Similar to the flexible TIP3P model, this flexible adaptation results in lower computational efficiency compared to the rigid TIP4P model and does not consistently yield superior outcomes.^30^

The Optimal Point Charge (OPC) is the first model formulated without geometric constraints^31^ from which, in turn, OPC3 has been derived. Analogously to OPC3, its parametrization is based on the reproduction of the actual electrostatic potential of water molecules, while removing limitations on point charge positioning. It represents a substantial advancement in accuracy over older water models.^31^

Water models with more than four points have also been parametrized. An increased number of interaction points may improve the representation of certain bulk properties of water. For instance, the Transferable Intermolecular Potential 5-Point (TIP5P) model can replicate the nonplanar charge distribution of water molecules. However, as *N* increases, the computational cost increases along with the number of parameters that need to be defined, while the associated improvements in accuracy are often not well-confirmed for biomolecular simulations.

Many other variations of the solvent models discussed here have been developed over the years with the aim of maximizing specific properties or include polarization effects. A comprehensive review can be found in ref (21).

When selecting a water model for biomolecular simulation, it is important to bear in mind that these models, commonly employed to represent aqueous solvation effects, have been traditionally parametrized at 298 K without considering any dissolved solute. Therefore, ensuring compatibility between the chosen water model and protein force fields is essential. For instance, TIP3P has been extensively used in combination with AMBER force fields, which have been fine-tuned for compatibility with the TIP3P water model.^22,23^ Recently, the AMBER team has advocated for the use of OPC water models alongside their latest biomolecular force fields. Differently, according to GROMACS recommendations, TIP4P is better suited in combination with the OPLS force field for proteins. However, it is important to highlight that the best fit between protein and water FFs for specific applications is often unknown.

Finally, a good water model should have the correct balance between accuracy and computational efficiency, as interactions among water molecules significantly impact the computational cost of simulations. Particularly, the computational expense of calculating interactions between water molecules correlates with the number of pairwise distances within the water model. Indeed, with 3-point models nine distances are needed for each pair of molecules, while ten are needed for 4-point models.

In this study, we systematically study how different water models affect the reconstruction of the Potential of Mean Force (PMF) from nonequilibrium simulations and the estimation of binding free energy. Unlike equilibrium simulations, nonequilibrium sampling methods such as SMD are greatly influenced by kinetics and energy barriers, affecting the amount of dissipated work obtained during the process. In this context, the kinetic and mechanical properties of the water model become crucial. This is especially true when dealing with small protein–ligand complexes with a limited number of degrees of freedom, where water is the main responsible of dissipation. Particularly, our work aims to study the effect of the most widely used water models on work dissipation and their suitability for nonequilibrium scenarios for two protein–ligand complexes, namely β-trypsin-benzamidine and glycogen synthase kinase 3 beta (GSK-3β) with its ATP-competitive inhibitor.

### 1.2 Path Collective Variables

When performing nonequilibrium SMD simulations, a pathway connecting the initial and final states of the system is followed. This requires the employment of a collective variable, which is a function of a few coordinates describing the evolution of the studied process. By applying nonequilibirum estimators to data from these simulations, the free energy profile along the used collective variable can be reconstructed. To support the CFT requirement for bidirectional SMD simulations, in our recent work^18^ we leveraged Path Collective Variables developed by Branduardi et al.^32^ PCVs are the ideal collective variable in this case as they describe the position of a point in the configurational space with respect to a predefined path, allowing to accurately follow the forward and backward evolution of the system.

The PCVs are *S*(*x*), measuring the progress along the path, and *Z*(*x*), measuring the orthogonal deviation from the path
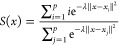
8
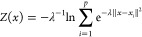
9Here, *p* is the number of molecular configurations in the path and  quantifies the distance between the *i*th conformation in the path (*x*_*i*_) and the instantaneous microscopic configuration (*x*), while λ is a smoothness parameter. *S*(*x*) represents a good mapping of the physical configurations as long as it is smooth and maps linearly the configurational space to the *S* progressive values. To obtain this faithful map one can parametrize λ with the following formula
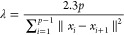
10where  quantifies the distance between the *i*th and the *i* + 1th configuration. This formula has been found heuristically by PCVs Authors.^33^

PCVs require the identification of a reference path to be followed during simulations. The approach used in our protocol to identify this reference path is detailed in the Methods section. This represents a critical step of the strategy, as the selected putative minimum free energy path strongly influences the results obtained. When a physically appropriate path describing the binding event is identified, then the free energy profile along the PCV *S*(*x*) and the binding free energy can be correctly estimated. The definition of a reference path that includes a mechanism different from the real binding one may result in very high energy barriers, which in turn result in high amount of dissipated work, preventing the accurate estimation of binding free energy.

In the following, we aim to understand how the candidate minimum free energy path and PCVs influence the reconstructed free energy profile along *S*(*x*) and the free energy estimate from nonequilibrium simulations. We focus on a small protein–ligand complex: T4 Lysozyme-*N*-phenylglycinonitrile. This complex, differently from previously discussed systems,^18^ presents a great challenge due to the deeply buried nature of the ligand within the protein.^34^ Finally, we discuss some guidelines to help the identification of an appropriate reference path based on the characteristics of the system.

## 2 Methods

To perform the SMD simulations with PCVs, reconstruct the Potential of Mean Force and estimate the binding free energy, we employed our recent computational strategy.^18^ SMD is a nonequilibrium method, thus nonequilibrium estimators are required to obtain the PMF and calculate the binding free energy. As discussed, our strategy relies on the bidirectional Crooks Fluctuation Theorem^8^ as this is more robust and provides faster convergence rates than the Jarzynski estimator. Nevertheless, here both estimators were used to understand the different production of dissipated work during binding and unbinding simulations.

In detail, our nonequilibrium SMD strategy is composed of five steps:

1. First, we devise a guess MD trajectory describing the unbinding event studied with the enhanced sampling method Adiabatic Bias MD (ABMD)^35^ coupled with an electrostatic-like CV^36^ to prompt protein–ligand dissociation. Several trajectories are run and the one with the smallest unbinding time and with the most plausible mechanism is selected as the initial guess path.
2. We identify a putative minimum free energy path of the unbinding event through the application of two path algorithms. Initially, the guess path is optimized in the configurational space from an initial bound state to a final unbound state using the Machine Learning principal path algorithm.^37^ Subsequently, the path is refined with an improved version of the Equidistant Waypoints Algorithm^38^ to render consecutive configurations of the path equidistant in terms of mean square deviation (MSD), as required for the correct definition of PCVs. As a result, a smooth path composed of consecutive equidistant molecular configurations capturing the (un)binding process is obtained. Finally, fictitious conformations generated through a short run of SMD are introduced at the beginning and end points of the path. While these conformations will not be reached during SMD simulations, they ensure a more consistent representation of the entire (un)binding process with PCVs, minimizing potential artifacts arising from PCV boundary conditions.
3. At this stage, nonequilibrium bidirectional SMD simulations with PCVs are run. SMD is a nonequilibrium method where a time-dependent harmonic potential *R*(*x*, *t*) is summed over the regular system potential *V*(*x*): 
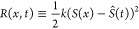
11Where *S*(*x*) is the currently observed value of the collective variable and *Ŝ*(*t*) is our pulling coordinate, progressing at constant speed in the simulation from the initial to the final state. Additionally, a harmonic wall (flat bottom potential) enables the confinement of the system along *Z*(*x*). During these irreversible simulations, the Jarzynski work (*W*_J_) values are collected to reconstruct the work profiles. Several binding and unbinding replicated simulations are performed until convergence of the binding free energy estimate is reached. Simulations for the systems used had a performance ranging from 80 to 120 ns/day using a dual socket node with Intel(R) Xeon(R) Silver 4210 CPUs and using 2 Tesla V100 GPUs.

4. Next, we reconstruct the PMF through the use of a nonequilibrium estimator via the automatic protocol devised.^18^ Our protocol relies on the Crooks Fluctuation Theorem^39^
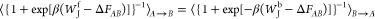
12In particular, considering the PCVs, we solve
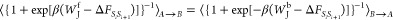
13where the PMF *F*(*S*_*i*+1_) is  and the *i* index represents the *i*-th configuration along the path. *W*_J_^f^ and *W*_J_^b^ are the work values from forward and backward simulations related to the *S*_*i*_, *S*_*i*+1_ interval. By solving eq 13 for increasing *i*, the PMF can be reconstructed. An analogous approach is used for the Jarzynski equality.

5. Lastly, the standard binding free energy is estimated as the sum of the binding free energy and the standard volume correction (as outlined by Doudou et al.^40^) 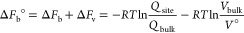
14Δ*F*_b_ is the ratio of the canonical partition functions of the bound (*Q*_site_) and unbound (*Q*_bulk_) states, computed by integrating the PMF
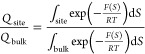
15To this end, a frame discriminating between two regions is determined via analysis of the PMF followed by visual inspection of trajectories. Then, the standard volume correction (Δ*F*_v_) is added to the binding free energy to define standard estimates, comparable with experimental values. Δ*F*_v_ represents the variation of the free energy due to considering the standard-state volume *V*° corresponding to 1661 Å^3^ (concentration of 1 M) instead of the effectively sampled unbound volume *V*_bulk_. This contribution is computed through NanoShaper^41^ and the BiKi software.^42^ Finally, binding free energy errors are calculated via bootstrap analysis with 500 bootstrap sampling.

### 2.1 Selected Systems

In order to study the impact of the water models and dissipation on the PMFs and the binding free energy estimate, we started by investigating the β-trypsin-benzamidine (TRY-BEN) binary complex, where the main interactions between the ligand and the receptor are electrostatic interactions and hydrogen bonding, leading to an experimental binding free energy of −6.2 kcal/mol.^43^ According to literature^34^ and our analysis, benzamidine is semiburied in the protein pocket.

We studied this system using both three-point models (TIP3P, TIP3P-flexible, OPC3) and four-point models (TIP4P, TIP4P-flexible, OPC). However, as we already investigated TRY-BEN using the water model TIP3P combining SMD, PCVs and the CFT, the reference path and the results in TIP3P were taken from our previous work.^18^ In this context, the complex was modeled starting from the crystallographic structure with PBD ID 3PTB using the force field AMBER99SB^44^ for Trypsin and GAFF^45^ (with AM1-BCC point charges, with a total charge of +1) for benzamidine. The system was solvated with TIP3P water molecules in a cubic box (12,847 solvent molecules), where the protein was placed at 1.2 nm from the box edges. Physiological salt concentration of 0.15 M and electric neutrality were achieved by adding sodium and chloride ions. Additional information regarding the energy minimization and equilibration processes can be found in ref (18). The final snapshot from the last equilibration step was used as the initial structure for *NVT* production runs. Here, in order to study the effect of the water model on binding free energy, the complex was solvated also using other water models: OPC3, TIP3P-flexible, TIP4P, OPC, TIP4P-flexible (12,847 and 12,777 water molecules for 3 and 4 points model respectively). Each solvated system was subjected to the same minimization and equilibration steps as the system modeled with TIP3P water model.

Subsequently, we confirmed the impact of water models on binding free energy using another system, involving glycogen synthase kinase-3 beta (GSK-3β), a system of pharmacological interest, and its ATP-competitive inhibitor 3-azanyl-*N*-(3-methoxypropyl)-6-[4-(4-methylpiperazin-1-yl)sulfonylphenyl]pyrazine-2-carboxamide for Alzheimer’s disease. In this complex, the protein pocket is shallow and exposed. The measured experimental binding free energy for this complex is −10.5 kcal/mol.^46^ Here, starting from the crystallographic structure with PDB ID 4ACH, the force field AMBER14SB^47^ was used to model the protein, while GAFF^45^ and AM1-BCC point charges (with a charge of +1 assigned to the nitrogen of the 4-methylpiperazine group) to model the ligand. The system was solvated with TIP3P water molecules in a cubic box. For the detailed protocol used to set up the GSK-3β system in TIP3P, we refer the reader to ref (36).

As for the TRY-BEN case, additional models of this complex were prepared using different water models, specifically the 3-point models OPC3 and the 4-point model TIP4P. Each system was solvated in a cubic box, where the protein was placed at 1.2 nm from the box edges (19,213 and 19,519 water molecules for 3 and 4 points model respectively). Electric neutrality was achieved by adding sodium and chloride ions. The fully solvated protein–ligand models were then subjected to energy minimization and equilibration following the same procedure as for TRY-BEN.

**Figure 1:**
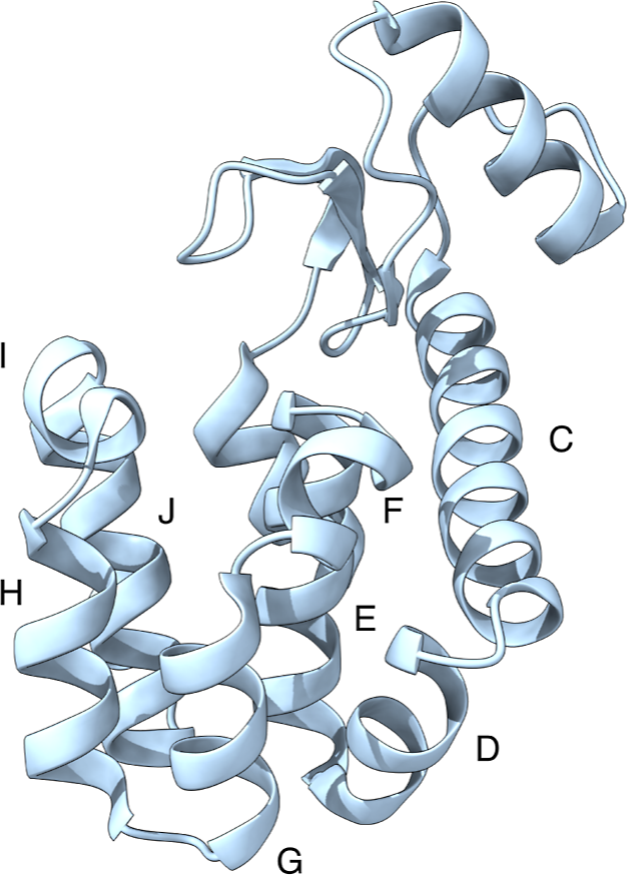
T4 Lysozyme crystal structure with PDB ID 2RBN. The helices in the C-terminal domain, involved in ligand binding, are labeled from C to H, as in ref (51).

Finally, we studied the impact that a reference path describing an incorrect mechanism can have on binding free energy estimations. Moreover, we analyzed how the inclusion of different degrees of freedom of the system in the reference path and the upper wall on *Z*(*x*) may affect the dissipated work during the nonequilibrium simulations, hence the convergence of results. For this aim, we investigated the T4 Lysozyme. T4 Lysozyme is composed of two domains: the N-terminal domain (from residue 1 to residue 70) and a barrel-shaped C-terminal domain (from residue 71 to 162) formed by 8 α-helices, where the ligand can bind (Figure 1). The T4 Lysozyme binding site (pertaining to the C-terminal domain) can exhibit different properties based on point mutations: the L99A binding site, which is apolar and does not contain water in the unbound state, and the L99A/M102Q site, a polar cavity that can form hydrogen bonds.^48^ Numerous studies have explored the potential pathways and structural transitions that small molecules use to escape from T4 Lysozyme L99A mutant. The substitution of leucine with alanine at position 99 in the wild-type T4 Lysozyme creates a 150 Å^3^ hydrophobic pocket within the protein C-domain. However, in this work, we focused on the less-studied L99A/M102Q double mutant, where the potential for hydrogen bonding is introduced by the M102Q substitution. The additional substitution of Methionine to Glutamine at position 102 provides a more polar engineered cavity site buried from bulk solvent. We chose a deposited structure of the T4 Lysozyme L99A/M102Q in complex with *N*-phenylglycinonitrile (PBD ID: 2RBN). This system is particularly challenging due to the ligand being deeply buried within the protein pocket.^34,49^ The main interactions in this protein–ligand complex are electrostatic ones and hydrogen bonding (hydrogen bond between amino N of the (phenylamino)acetonitrile and Gln102 of the protein), leading to an experimental binding free energy of −5.52 ± 0.18 kcal/mol.^43^

Starting from the crystallographic structure, the complex was modeled using the force field AMBER99SB^44^ for the lysozyme and GAFF (with AM1-BCC point charges, with a total charge of 0) for the (phenylamino)acetonitrile ligand. Since several side chains of lysine residues were missing in the original crystallographic structure deposited in the PDB, preliminary modeling was performed using the Swiss Model web tool.^50^ Following the same strategy used for the other systems, the complex was solvated with the TIP3P water model, neutralized and finally equilibrated.

## 3 Results and Discussion

During nonequilibrium simulations dissipation arises, resulting in irreversible processes with an average work greater than the free energy difference. The excess work is referred to as dissipated work (eq 2). As mentioned, minimizing dissipation is a central optimization problem for our nonequilibrium protocol. To address this, we conducted a systematic study focusing on two key factors contributing to dissipation: the choice of water model and the definition of PCVs, obtaining in the end a protocol that minimizes dissipation in those two respects.

### 3.1 Dissipation due to Water Model

In this section, we investigated the dependence of dissipated work due to the water model employed in nonequilibrium simulations. Specifically, we evaluated the dissipated Jarzynski work (eq 2) for binding and unbinding simulations by changing the water model. This work is defined as the difference between the average final cumulative Jarzynski work (eq 1) obtained from SMD and the converged estimate with the CFT, .

For this systematic analysis, we examined the TRY-BEN and GSK-3β complexes. In the case of TRY-BEN, a reference path was previously obtained for the system solvated with the TIP3P water model.^18^ We assumed that this path remains unchanged across different water models. This assumption is justified because the path was built from unbinding simulations, where the displacement of water networks is not the critical event (in contrast to binding simulations, which might lead to slight differences due to the water model). Consequently, the same path obtained with TIP3P was employed in all replicated bidirectional SMD simulations, regardless of the specific water model used.

For the GSK-3β system, we defined a reference path consisting of 118 equidistant conformations of the ligand describing the unbinding event (plus the fictitious conformations of the end-points). This path was derived from an electrostatic-like Adiabatic Bias MD unbinding simulation of the system solvated with TIP3P water. Assuming the path does not change due to the water model, as for TRY-BEN, this path was used in all the bidirectional simulations replicas of SMD with different water force fields.

To assess the impact of water models and steer velocities on the binding free energy estimates, bidirectional simulations were run for three different simulation times: 10, 50, and 100 ns. The SMD setup is described in ref (18). In order to reach a stable and converged estimate with respect to the number of replicas, respectively 50, 30, and 20 replicas were required for TRY-BEN, while 30, 25, and 15 for GSK-3β. These correspond to total simulation times up to 4 and 3 μs, respectively. We remark that these simulations can be run in parallel.

The work profiles obtained from SMD simulations at different pulling speeds are reported in Figures S1–S4 for TRY-BEN and Figures S5–S8 for GSK-3β.

**Figure 2:**
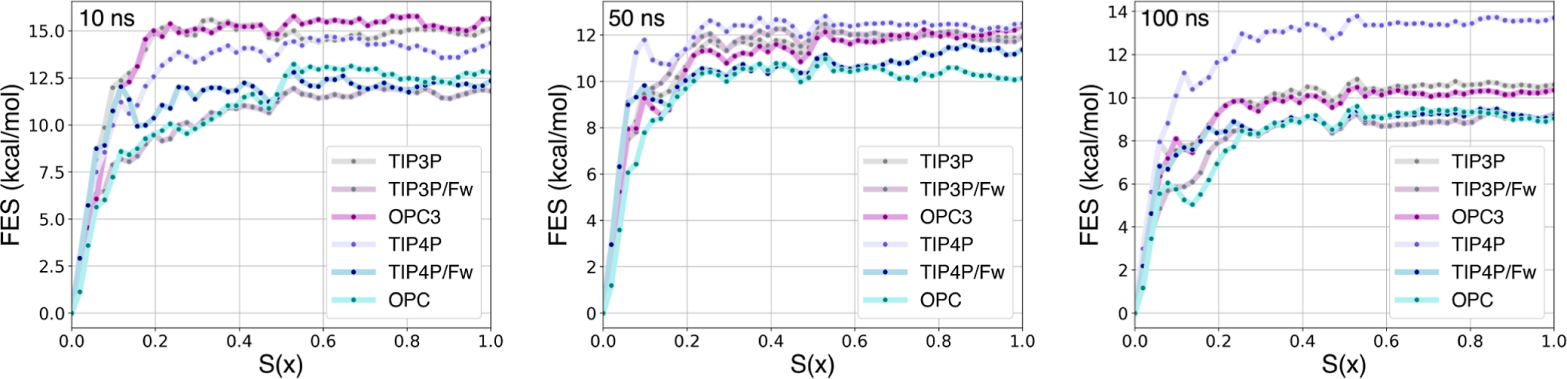
Free energy profiles along *S*(*x*) [*S*(*x*) = 0 bound state and *S*(*x*) = 1 unbound state] obtained by applying the CFT to varying time length SMD simulations of the TRY-BEN system solvated with different water models.

**Figure 3:**
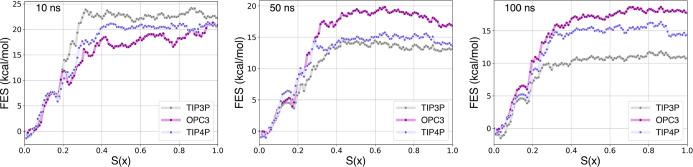
Free energy profiles along *S*(*x*) [*S*(*x*) = 0 bound state and *S*(*x*) = 1 unbound state] obtained by applying the CFT to varying time length SMD simulations of the GSK-3β system solvated with different water models.

Relying on our protocol and the nonequilibrium estimator CFT, we reconstructed the corresponding free energy profiles along *S*(*x*), where *S*(*x*) = 0 corresponds to the bound state and *S*(*x*) = 1 to the unbound state. Figure 2 presents the free energy profiles along *S*(*x*) obtained via the CFT for TRY-BEN, while Figure 3 for GSK-3β.

By computing the ratio of the bound and unbound regions partition functions (eq 15), we estimated the binding free energy. To this end, we defined a value of *S*(*x*) discriminating between the two regions. As suggested by Doudou et al.,^40^ this value corresponds to the point where the PMF becomes constant within statistical noise and the ligand becomes solvated. Considering this criterion and visual inspection of the trajectories, we selected the 8th configuration of the path for TRY-BEN and the 35th configuration for GSK-3β, both related to a partially solvated state. Finally, the binding free energies calculated via the ratio of partition functions were refined with the standard volume correction (eq 14), yielding standard binding free energies (Δ*F*°), comparable to experimental binding affinities.

For completeness, we compared the results of the bidirectional estimator with those of the unidirectional Jarzynski estimator. The standard binding free energies estimated with the Jarzynski estimator are reported in Table S1 for TRY-BEN and in Table S2 for GSK-3β.

In order to evaluate the effectiveness of the method, both the convergence of the estimates (variance) and their accuracy (bias) are considered. Specifically, convergence is assessed by analyzing the stability of the estimates as the simulation time varies, while accuracy is estimated based on the recovery of the experimental value upon convergence.

#### 3.1.1 Impact of Water Models on Trypsin-Benzamidine

The unbinding work profiles of TRY-BEN (Figures S1 and S3) illustrate the increase of the cumulative *W*_J_ over time, reaching a plateau when the unbound state is achieved. As expected, for all water models, the cumulative *W*_J_ decreases as the total simulation time increases, since lower steering velocities result in lower dissipated work. For binding simulations (Figures S2 and S4), *W*_J_ initially remains close to zero as the ligand is solvated and is expected to become increasingly negative over time (as the sign of Δ*F* is changed for consistency with unbinding simulations). However, for each water model, only for sufficiently long SMD simulations the profiles exhibit a decrease in the dissipated work.

The effect of different water models on the work profiles becomes less pronounced in longer simulations. Indeed, the 100 ns SMD simulations for various water models show greater similarity in terms of dissipated work. In contrast, in faster 10 ns SMD simulations, kinetics plays a crucial role and strongly affects the dissipated work, increasing the influence of the water model. In particular, more significant differences in the work due to the water model are observed in fast binding simulations. During binding, the ligand must displace water molecules from the pocket to reach the bound state, making this event heavily dependent on the behavior of water molecules.^52^ The more difficult it is for the ligand to displace the water molecules, the higher the resulting *W*_J_. Contrarily, the unbinding event is largely dominated by the protein behavior and the work is mainly generated by the force required to disrupt the ligand interactions with the protein and pull it out of the binding pocket.

Analogous observations can be made for the potentials of mean force obtained using the CFT estimator (Figure 2). Local heat production changes based on the water model employed, strongly affecting the dissipated work and, hence, the PMF. As mentioned, this is especially noticeable in fast simulations, where the more prominent role of kinetics amplifies differences among water models. Indeed, the PMFs and the standard binding free energy estimates obtained with the CFT from 10 ns simulations vary significantly depending on the water model employed, as shown in Figure 2 and Table 3. Differently, PMFs from longer SMD simulations exhibit a higher degree of consistency across various water models, with CFT estimates converging to comparable standard binding free energy values (Table 3). The only exception is the TIP4P model, which deviates considerably from the others. While estimates from other water models exhibit convergence toward the experimental value as the total simulation time increases, TIP4P estimates seem rather invariant to different pulling speeds, demonstrating low variance across the simulation times tested. Consequently, the TIP4P CFT results quickly converge to a stable value (even for fast 10 ns simulations), in contrast to the 3-point models TIP3P and OPC3, which require 100 ns simulations to achieve convergence of the estimates. Nevertheless, with the TIP4P model, a significant bias in the estimates is observed, strongly deviating both from the experimental and the converged estimates of the other water models. Despite the TIP3P and OPC3 models requiring longer simulation times to reach convergence, they lead to estimates with minimal bias, closely aligning with experimental values. In contrast, although OPC is considered one of the best available water models,^31^ its estimates in this nonequilibrium protocol and system are strongly affected by both variance and bias, leading to overall unsatisfactory results. Finally, the flexible water models investigated here did not show substantial bias, yielding estimates that are in reasonable agreement with the experimental value. Despite these results being comparable to TIP3P and OPC3 results, the inherent higher computational cost required by flexible models makes them less attractive options compared to TIP3P and OPC3.

**Table 3:** Standard Binding Free Energies (kcal/mol) for TRY-BEN Estimated Using the CFT for Increasing Simulation Times Compared to the Experimental Affinity

model	10 ns	50 ns	100 ns
TIP3P	–12 ± 1	–8.1 ± 0.5	–6.4 ± 0.8
TIP3P/Fw	–6.9 ± 0.9	–8.6 ± 0.7	–5.1 ± 0.8
OPC3	–11.6 ± 0.9	–7.5 ± 0.5	–6.5 ± 0.8
TIP4P	–9.7 ± 0.8	–9 ± 1	–9.2 ± 0.6
TIP4P/Fw	–8.4 ± 0.7	–7 ± 1	–5.9 ± 0.7
OPC	–7.5 ± 0.8	–7.3 ± 0.4	–4 ± 1
experimental	–6.2		

These observations for the TRY-BEN complex motivated us to further investigate the impact of the TIP3P, OPC3, and TIP4P models on a more challenging system.

#### 3.1.2 Impact of Water Models on GSK-3β

The work profiles for the GSK-3β system, obtained at different pulling speeds and with different water models, are reported in Figures S5–S8. Similar considerations to those made for TRY-BEN can be drawn.

By analyzing the PMFs (Figure 3) and the binding free energy estimates (Table 4), we found that the TIP3P simulations exhibit slower convergence and higher variance compared to other models, as the TIP3P PMFs and estimates fluctuate significantly with the simulation time. However, similarly to the TRY-BEN case, the TIP3P estimate for GSK-3β at 100 ns is less biased and in good agreement with the experimental value (−10.5 kcal/mol). On the other hand, the OPC3 and TIP4P models exhibit faster convergence (hence, lower variance) and their estimates stabilize at 10 and 50 ns, respectively. Nevertheless, their converged estimates have a higher bias also at 100 ns, deviating more from the experimental value compared to the 100 ns TIP3P estimate. The standard binding free energies determined with the Jarzynski estimator are reported in Table S2. Lastly, we observe that for both TRY-BEN and GSK-3β the binding event is substantially barrier-less. In the case of TRY-BEN this is mainly due to the small size of the ligand, whereas in the case of GSK-3β, the goodness of the ABMD-derived path and the solvent exposed nature of the site, render the binding a smooth process.

**Table 4:** Standard Binding Free Energies (kcal/mol) Estimated Using the CFT for GSK-3β Increasing Simulation Times Compared to the Experimental Affinity

model	10 ns	50 ns	100 ns
TIP3P	–22 ± 3	–12 ± 1	–10.7 ± 0.7
OPC3	–16 ± 3	–17 ± 3	–16 ± 1
TIP4P	–19 ± 2	–13.8 ± 0.6	–14 ± 1
experimental	–10.5		

#### 3.1.3 Discussion on Water Model Dissipation

Technically, all the investigated water models are compatible with the protein and ligand force field used (i.e., AMBER and GAFF). However, protein force field parametrization is typically performed with a specific water model only. Therefore, the accuracy of the predicted biomolecular properties may be influenced by the specific water and protein force fields. Additionally, the selection of ion parameters for neutralization is crucial and should match the chosen water model, as we performed in our study to ensure consistency and reliability in simulation outcomes.

In this work, dissipation is evaluated from two perspectives for each water model: (i) in terms of cumulative dissipated work during 100 ns simulations and (ii) in terms of variation in dissipation over the total simulation time.

**Figure 4:**
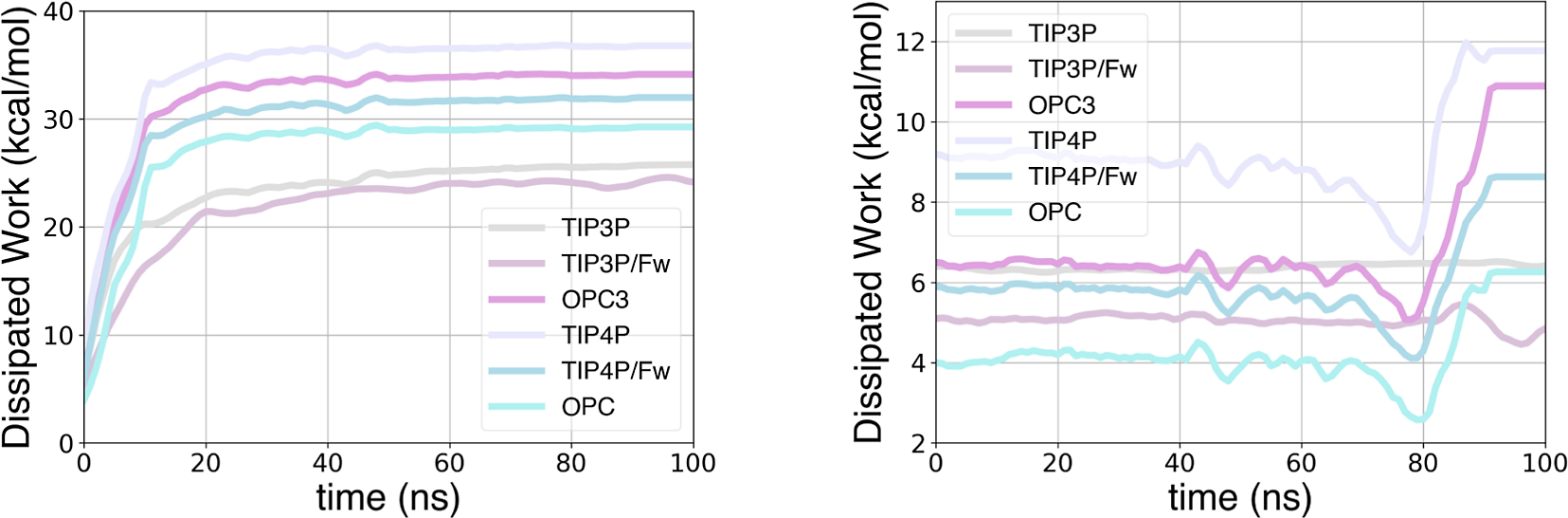
Evolution of dissipated work during 100 ns binding (left) and unbinding (right) simulations for TRY-BEN with different water models.

**Figure 5:**
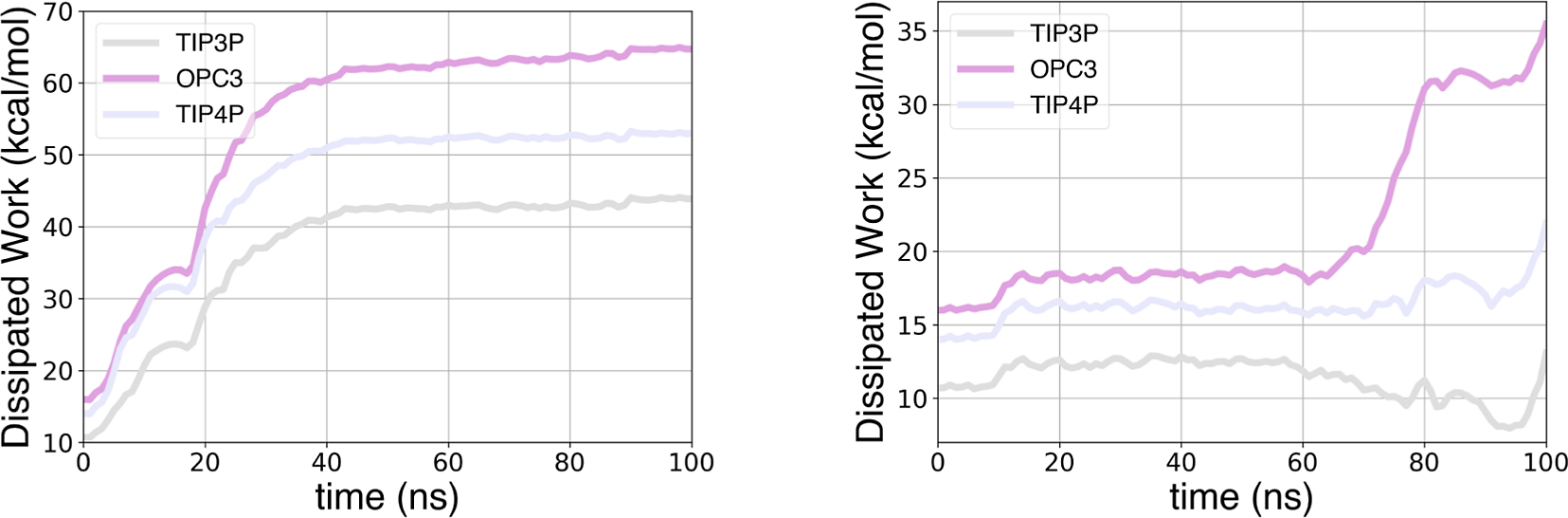
Evolution of dissipated work during 100 ns binding (left) and unbinding (right) simulations for GSK-3β with different water models.

The variation of cumulative dissipated work during both binding and unbinding 100 ns simulations is reported for TRY-BEN in Figure 4 and for GSK-3β in Figure 5. These plots illustrate how dissipation evolves over time during the 100 ns simulations. For the 100 ns unbinding simulations, the dissipation is rather similar among water models; although TIP3P, in both flexible and rigid versions, demonstrates the lowest dissipation. On the other hand, during 100 ns binding simulations, there is an increase in dissipation toward the end of the simulations, coinciding with the binding. This occurs because when the ligand becomes partially bound and attempts to displace water molecules from the pocket, a significant amount of work is generated. The increase in dissipation is more pronounced when it is more difficult for the ligand to displace the water molecules, as can be seen with the OPC3, OPC, and TIP4P models. In contrast, the dissipation increase is lower with TIP3P/Fw and TIP4P/Fw, and is almost absent when using TIP3P, where the dissipated work remains relatively stable.

**Figure 6:**
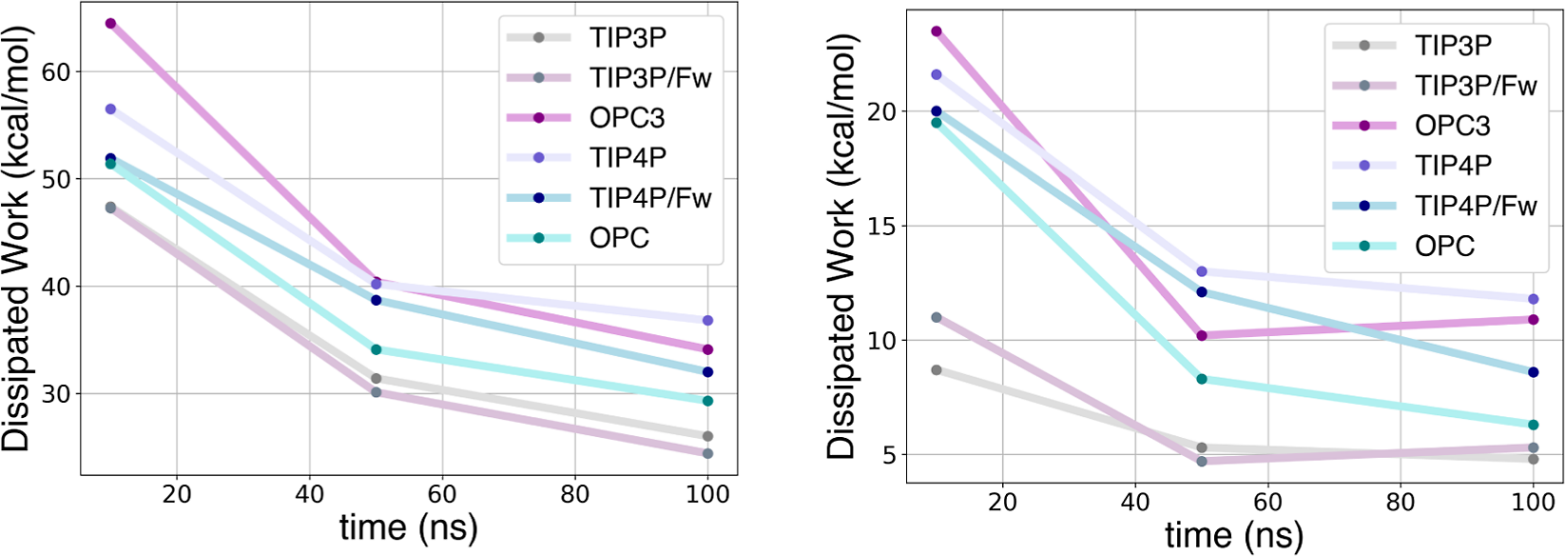
Dissipated work vs total simulation time for unbinding (left) and binding (right) simulations of TRY-BEN with various water models.

**Figure 7:**
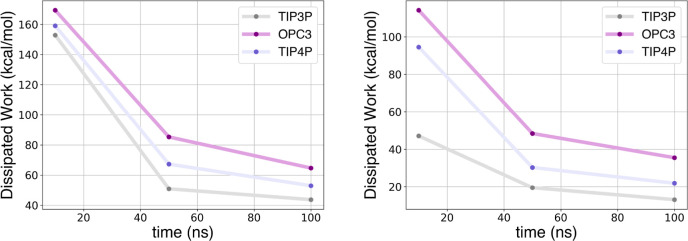
Dissipated work vs total simulation time for unbinding (left) and binding (right) simulations of GSK-3β with various water models.

The variation in dissipation over increasing total simulation times is reported for TRY-BEN in Figure 6 and for GSK-3β in Figure 7. These plots display the capacity of different water models to reduce dissipation when increasing the total simulation length. This provides a measure of the efficiency of each water model in mitigating dissipation. The steeper the work reduction in the plot, the greater the capacity of the water models to facilitate convergence. However, as previously highlighted, fast convergence does not always result in an accurate estimate. All models benefit from longer simulation time for both systems analyzed, demonstrating the advantages of using longer simulation times. By computing the derivative of the dissipated work with respect to the total simulated time, we found that the 4-point water models investigated here are more significantly influenced by longer simulation time for both systems. On the other hand, despite TIP3P is not the most positively affected by longer simulations, it consistently shows the lowest overall dissipation.

For both systems, our results indicate that the TIP3P water model is the most appropriate when aiming to reduce dissipation in nonequilibrium simulations and achieve an accurate estimate.

This can be attributed to the lower viscosity of TIP3P water compared to the other models analyzed, which positively impacts the work dissipation during water displacement. A possible explanation of this lies in the parametrization strategy originally used for these water models. TIP3P parametrization process did not account for the self-diffusion coefficient, resulting in a higher value than the experimental one. The other models investigated, instead, have a lower self-diffusion, closer to the experimental value. This discrepancy translates into stronger hydrogen bonds and higher viscosity for TIP4P, OPC3, and OPC, while TIP3P shows weaker hydrogen bonds and lower viscosity. The viscosity of water significantly influences the interactions between water molecules and solutes. In nonequilibrium binding simulations, it affects the ability of the ligand to get in proximity of the pocket and displace water molecules from the pocket to reach the bound state. Since TIP3P hydrogen networks are weaker, these events will be eased, leading to lower dissipation. An additional key parameter that influences the viscosity of water is the partial atomic charges of water molecules. The 4-point water models analyzed here exhibit higher partial atomic charges in absolute values than the 3-point models, resulting in higher viscosity for 4-point models. Among the investigated models, OPC has the highest absolute partial charges, while OPC3 has the highest among 3-point models. Consequently, these models present enhanced viscosity. Despite the viscosity of OPC and OPC3 being closer to the experimental value than TIP3P and TIP4P, this results in higher dissipated work in nonequilibrium simulations.^23^ Moreover, when electrostatic interactions are relevant (as in the case of TRY-BEN and GSK-3β), the dielectric constant of water plays an important role. This parameter is directly linked to the strength of water–water interactions: the higher the dielectric constant, the weaker the electrostatic interactions. For TIP models, this quantity was not considered during parametrization, resulting in a strong overestimation of the dielectric constant for TIP3P and an underestimation for TIP4P compared to the experimental value. As a result, TIP4P has stronger electrostatic interactions, affecting hydrogen networks and the displacement of water molecules. This again explains the higher viscosity of TIP4P and its associated greater dissipation compared to TIP3P. The experimental values of the main properties of water molecules compared to the values calculated with the force fields discussed here are reported in Table S3.

In conclusion, the combination of the Amber protein force field and GAFF for the ligand with the TIP3P water model offers the best combination for nonequilibrium scenarios. The TIP3P model is the least affected by work dissipation and its estimates have a low bias, making it the most advantageous choice for achieving good agreement between experimental results and calculations in nonequilibrium simulation with a feasible computational cost. Despite not being the latest conceived water model nor ideal in accurately reproducing hydrogen bonding networks, its lower viscosity makes TIP3P the most suitable option for nonequilibrium simulations from a practical perspective.

Additionally, it is important to highlight that the superior performance of TIP3P compared to more refined water models may also be attributed to its use during the parametrization process of GAFF.^53^

### 3.2 Dissipation due to PCVs

In this section, the goal is to understand the impact that PCVs have on the Jarzynski work obtained during SMD simulations. For the TRY-BEN and GSK-3β systems investigated here and the previously discussed CB8-G8 toy model,^18^ the definition of PCVs with our protocol was rather straightforward and required no special considerations. This was due to the reduced degree of complexity of these systems, as they were small and medium-sized and had a completely or rather shallow binding pocket. Moreover, they had no relevant degrees of freedom orthogonal to those handled by PCVs.

However, when we applied our pipeline to more intricate systems, displaying relevant protein rearrangements, we encountered some difficulties in defining suitable PCVs. This was reflected in the high values of Jarzynski work, thus in a considerable challenge in obtaining satisfactory results compared to the experimental affinity. To reduce this effect, longer SMD simulations can be run to allow for the relaxation of all relevant degrees of freedom. However, for complex systems, the computational cost of this approach would be excessively high.

To efficiently extend our computational pipeline from benchmark to real pharmaceutical systems, it is important to fine-tune some of its steps. In particular, in this section, we analyzed key components of the pipeline: the reference path and PCVs. Our aim was to understand how PCVs influence the work dissipated in nonequilibrium simulations of complex systems and attempt strategies to reduce it, favoring the convergence of binding free energy estimators.

To this end, we selected the T4 Lysozyme complexed with the *N*-phenylglycinonitrile ligand, focusing specifically on the less-explored L99A/M102Q double mutant. This choice was motivated by its highly buried pocket, which provides an ideal context to investigate the effects of binding pathways and PCV definitions on dissipation. Additionally, this system offered the opportunity to analyze the orthogonal deviations from the path during SMD simulations, as there is no single well-established unbinding pathway for this complex.

Also for the T4 Lysozyme system, we expect a dependence of results on the water model employed during nonequilibrium SMD simulations. However, considering the results obtained in the previous section, we believe that the less viscous TIP3P model is ideal for this deeply buried system, allowing the binding pocket to be more easily desolvated when the ligand binds.

The results for T4 Lysozyme complexed with *N*-phenylglycinonitrile ligand are discussed in the following. Accordingly, we will provide guidelines on defining PCVs tailored to the characteristics of the system studied, in order to improve the convergence of our nonequilibrium protocol.

#### 3.2.1 Reference Path Definition

The identification of a minimum free energy path would lead to a low-dissipation pathway. Conversely, defining a reference path that includes a mechanism different from the real, most probable, binding mechanism may result in very high free energy barriers encountered during simulations, causing high amounts of dissipated work and preventing the accurate estimation of binding free energy.

As mentioned, the systems previously studied have superficial or semisuperficial binding pockets, so the identification of an unbinding path resulted quite simple. However, for systems with a deeply buried binding pocket, such as the T4 Lysozyme complex with *N*-phenylglycinonitrile, identifying the most probable exiting pathway of the ligand can be challenging.^34,49,54^ Buried protein cavities can often trap water molecules. Therefore, the exchange of water between the bulk solvent and these cavities can be particularly slow, as it may require significant conformational changes in both the protein and the solvent network to facilitate water escape.

**Figure 8:**
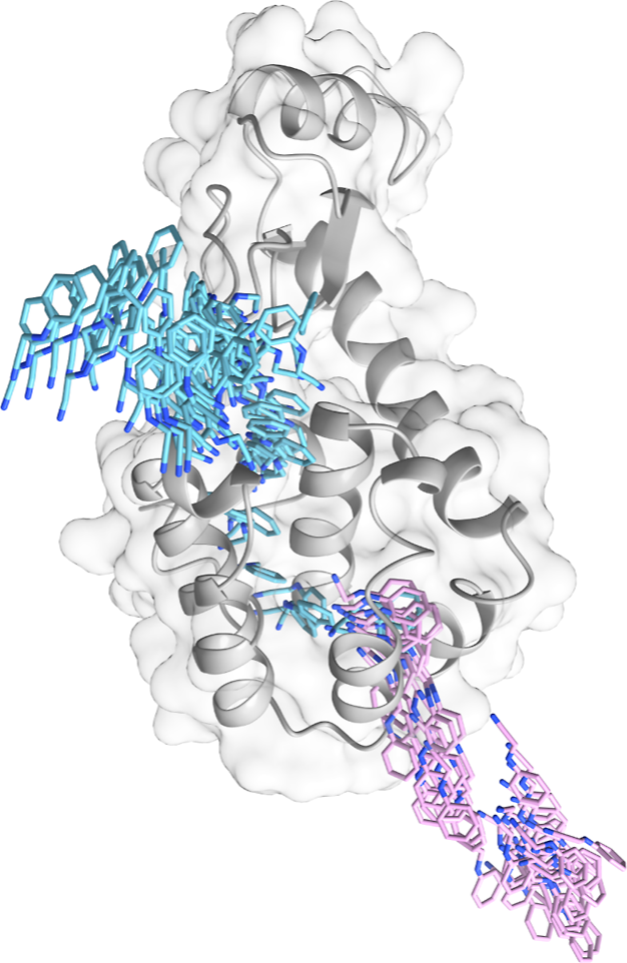
Reference paths obtained for T4 Lysozyme in complex with *N*-phenylglycinonitrile: Path 1 in pink and Path 2 in light blue.

As the first step of our protocol is sampling the ligand exit, we performed multiple unbinding ABMD simulations of 10 ns each with the TIP3P water model. Even though ABMD is a rather gentle method,^35^ it still requires long simulation times to accurately capture water exchange both inside and outside the binding cavity, as well as the necessary conformational rearrangements for buried ligands and flexible proteins. This complexity often makes it challenging to identify a single predominant path. In contrast to cases previously studied, where a single well-defined exit path was identified, the ABMD trajectories of this system revealed the presence of multiple escape routes. In T4 Lysozyme, the binding pocket is deeply buried, with no distinct tunnel leading to the protein surface (also according to our NanoShaper analysis^41^). As a result, the protein must undergo structural fluctuations or conformational “breathing” to permit the ligand to access/exit the binding site.^49^ Moreover, as we considered a structure with two-point mutations (L99A and M102Q), the cavity is polar, posing additional complexities due to the need to correctly orienting molecules for hydrogen bonding to Gln102. From our analysis, two main unbinding pathways were found, which we will refer to as Path 1 and Path 2. Although guess Path 2 is the most likely to occur, the other path also has a not null probability of occurring. Therefore, we applied the path algorithms to both, resulting in two final reference paths, as shown in Figure 8. Path 1 and Path 2 consist solely of ligand atoms and are composed of 49 and 73 equidistant conformations (with C_α_ of the protein pocket for alignment before computing the MSD), respectively, representing the possible ligand (un)binding pathways.

In Path 1 (in pink in Figure 8), the ligand moves through the lower part of helices G and D, and a similar path has been reported also for the L99A mutant.^54^ Meanwhile, in Path 2 (in light-blue in Figure 8), it exits through helices G and D but closer to helix F, requiring a significant rearrangement of the upper part of the buried binding site. Thus, significant motions of helix F (residues 108–114) are observed in this case to enlarge the cavity and facilitate ligand unbinding.

We highlight that the scope of the ABMD simulations was to identify meaningful pathways from a mechanistic point of view. In particular, we were interested in pathways associated with low energetic barriers, hence a low dissipation, which would lead to faster convergence when used in free energy calculations. Although simulations revealed only two main paths compatible with these criteria, other binding and unbinding pathways may be identified for the T4 Lysozyme L99A/M102Q mutant.^51,54^

Following our protocol, we performed 25 binding and 25 unbinding PCVs-SMD simulations of 100 ns for each reference path determined, for a total simulation time of 5 μs for each reference path. Subsequently, we reconstructed the Jarzynski work profiles for each series and we applied the CFT estimator. Finally, we corrected these estimates with the volume correction (eq 14), obtaining standard binding free energies (Δ*F*_b_^°^) that can be compared with the experimental value of −5.52 ± 0.18 kcal/mol.

For Path 1, from the work curves obtained (Figure S9), we estimated the PMF and computed the Δ*F*_b_^°^ estimate, resulting in −4 ± 1 kcal/mol. Moreover, to understand the dissipation obtained, we computed the dissipated work for both forward and backward transformations (eq 2) as the difference between the average *W*_J_ and the CFT estimate obtained, . The binding free energy and the related work dissipation for Path 1 are reported in Table 5.

**Table 5:** Standard Binding Free Energies (kcal/mol) Estimated Using the CFT and Dissipated Work Values (kcal/mol) for 100 ns SMD Simulations of the T4 Lysozyme Using Different Paths^a^

path	Δ*F*_CFT_	*W*_diss,bind_	*W*_diss,unbind_
Path 1	–4 ± 1	32	40
Path 2			
Path 1 with pocket	–2 ± 4	55	29
Path 2 with pocket	–8 ± 2	29	58
Path 1 with pocket and smaller Z	–7.8 ± 0.9	8	32

a Values for Path 2 are missing as the high dissipated work prevented their calculation.

**Figure 9:**
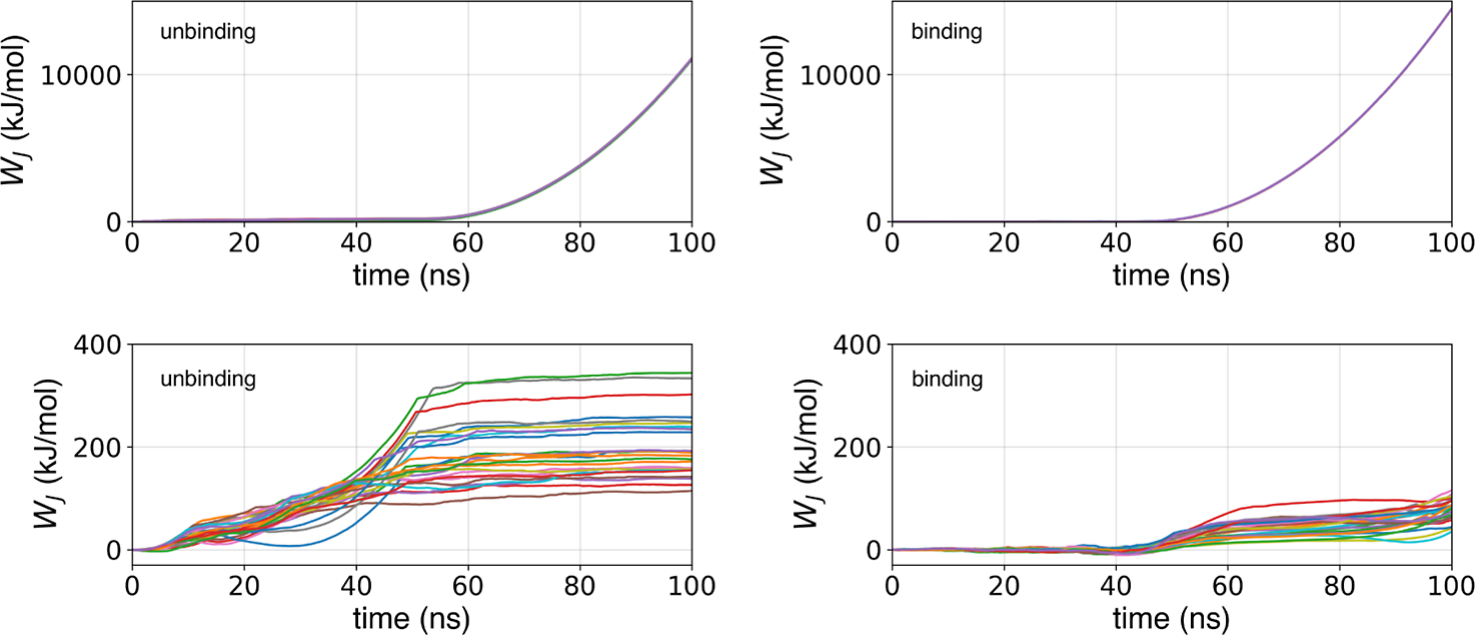
Jarzynski work profiles for unbinding and binding simulation of 2RBN using Path 2 with only ligand atoms (upper section) and refined Path 2 including ligand and pocket atoms (lower section).

Differently, examining the work curves of Path 2 (upper section of Figure 9) an unexpected behavior is revealed. When the system assumes a partially unbound conformation (*S*(*x*) in the proximity of 39 and 40), the ligand remains attached to the surface, unable to transition toward the (un)bound state in (un)binding simulations. The trajectories show that the ligand is trapped due to several interactions with side chains of amino acid residues pertaining to helix F and its neighbors (particularly, Arg125, Arg119, Asn116, Met120, and Gln123). These interactions with the surface prevent the ligand from synchronously following the moving SMD center. Consequently, the Jarzynski work (which depends on the distance between the ligand coordinates and the center of the bias) will increase exponentially, making it impossible to compute the binding free energy.

This behavior can be attributed to the fact that Path 2 needs a significant rearrangement of the pocket, particularly involving helices F and G, to allow for the ligand to fully exit the pocket. This highlights the critical role of pocket rearrangements in systems with buried binding sites. All of these findings were further evaluated and confirmed through Well-Tempered MetaDynamics analysis, as detailed in Supporting Information.

The issues encountered emphasize the need to modify our approach for the reference path definition. In the original strategy, successful on simple systems, its definition involved only ligand atoms (with C_α_ of the protein pocket used only for alignment before computing the MSD). Nevertheless, when complex and large systems are treated, this may result in significant amounts of dissipated work, despite the fact that the ligand path is apparently mechanistically accurate. This is because there may be several degrees of freedom of the protein not included in the reference path, but relevant to the mechanism. In this case, the SMD bias will only pull the degrees of freedom included in the path (related to the ligand atoms), thus only the ligand will evolve during (un)binding according to the reference path, while the protein may behave differently to the actual mechanism. Therefore, protein degrees of freedom that are relevant to the (un)binding process must be included.

To correctly describe the relevant protein rearrangements, we included the protein pocket atoms along with the ligand ones in the reference path definition. With this enhancement, during binding SMD, the bias potential not only pulls the ligand toward the binding pocket, but also facilitates the transition of the protein pocket from a close to an open conformation or vice versa. Starting from the same ABMD trajectory used to generate the previous paths, we used the refined procedure to generate new reference paths that include both ligand and pocket heavy atoms (within 6 Å from the ligand). The new reference paths are composed of 20 and 29 conformations for Path 1 and Path 2, respectively.

By using these refined paths (also including pocket atoms), we performed 25 binding and 25 unbinding replicas of bidirectional SMD. The work profiles obtained with this path modification demonstrate that the introduction of pocket atoms in Path 2 was very beneficial, leading to a strong reduction of the generated work (lower section of Figure 9). This enabled us to estimate the binding free energy with the CFT for both paths including the pocket. These values are reported in Table 5, along with the dissipated work calculated for both paths with respect to their converged CFT estimate.

The dissipation and binding free energy values for the different path definitions show differences. This demonstrates that the results for this challenging system are highly sensitive to the definition of the PCVs.

**Figure 10:**
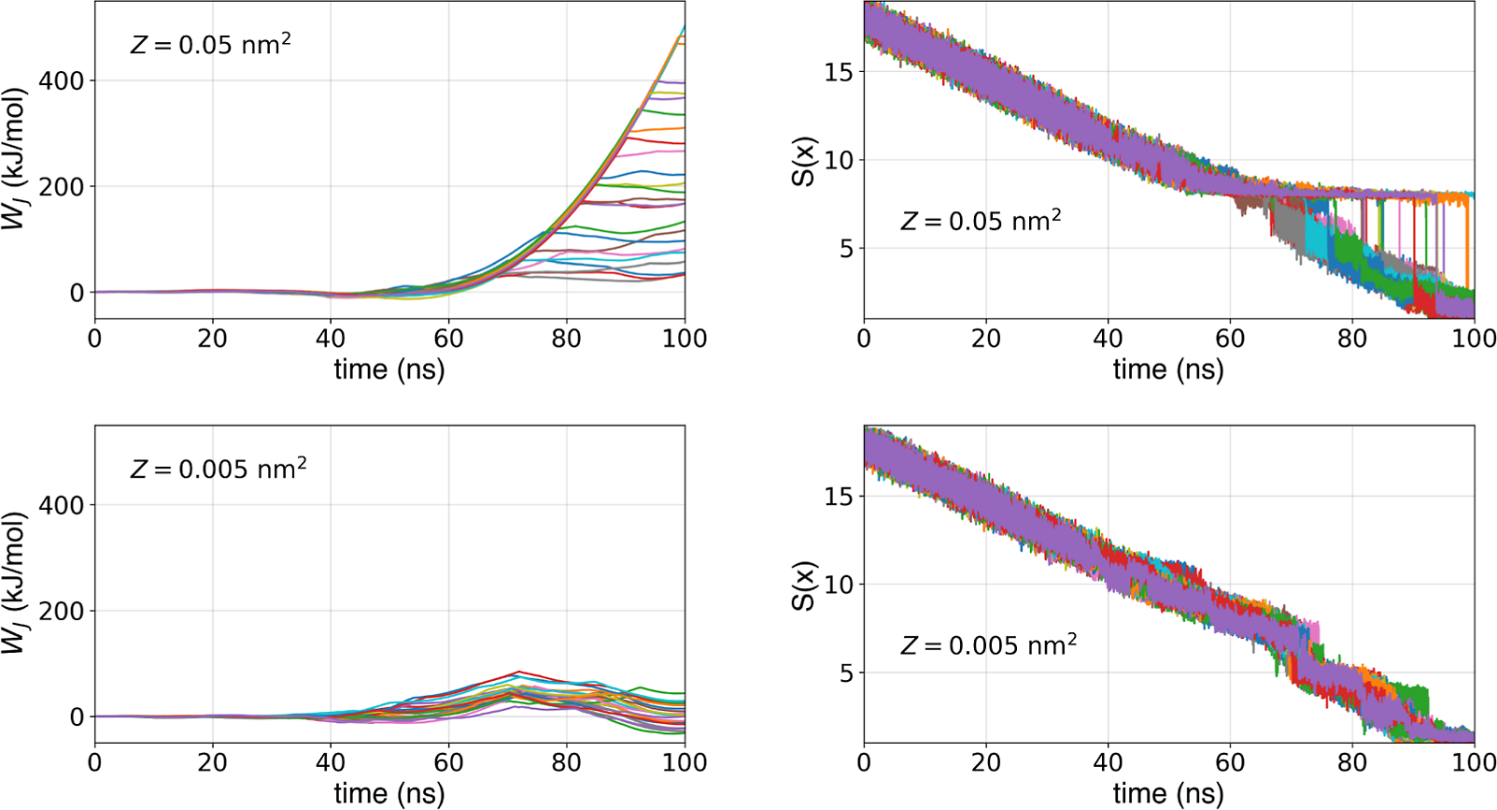
Jarzynski work profiles and corresponding evolution of *S*(*x*) for binding simulation of 2RBN using Path 1 with ligand and pocket atoms with an upper wall centered on *Z* = 0.05 nm^2^ (upper section) and *Z* = 0.005 nm^2^ (lower section).

While the inclusion of the pocket improves the results with Path 2, the same does not hold true for Path 1. For this new version of Path 1, the dissipated work during binding presents an increase compared to the initial Path 1, leading to a binding free energy estimate further away from the experimental value. Indeed, the binding work profiles with the new version of Path 1, illustrated in the upper section of Figure 10 (unbinding profiles in Figure S11), present a marked increase in *W*_J_ when reaching the bound state.

#### 3.2.2 Orthogonal Deviation from the Path

Despite refining the PCVs, the results remain unsatisfactory for Path 1. Visual inspection revealed that for some binding trajectories the PCV *S*(*x*) has difficulty tightly following the center of the SMD bias *Ŝ*(*t*) (which evolves linearly with time), as highlighted in the upper section of Figure 10. Consequently, in these trajectories, the system is not in the stiff-spring approximation, i.e., the progression of *S*(*x*) is not synchronized with that of *Ŝ*(*t*).

First, one might expect this problem to be due to a reduced spring stiffness and that increasing it would ensure that the system closely follows *Ŝ*(*t*).^55^ However, this is not the case. In fact, high values of *k* lead to very high values of *W*_J_, introducing undesired dissipation.

In order to address this, we must ensure that the evolution of the system follows the evolution of the SMD center, hence the reference path, without becoming trapped on the protein surface. To this end, our strategy is to act on the orthogonal deviation of the system with respect to the path, i.e., on the PCV *Z*. During our SMD simulations, to avoid strong deviations in the system evolution from the reference path, an upper wall along *Z* is used. Hitherto, the threshold of the upper wall has been *Z* = 0.05 nm^2^, i.e., only when the orthogonal deviation of the system *Z*(*x*) surpassed *Z* = 0.05 nm^2^ the harmonic restraint is active. This value was selected to guarantee the correct progression of trajectories while ensuring ligand conformational rearrangements. Nevertheless, in this specific case, a harmonic wall centered on *Z* = 0.05 nm^2^ appears to be too loose, allowing the ligand to deviate significantly from the reference path. Consequently, when such problems are encountered, our suggestion is to reduce the value at which the wall on *Z* becomes active.

Bidirectional SMD simulations were run again for T4 Lysozyme system using the same Path 1 (hence, without modifying *S*) but this time with the threshold of the upper wall set to *Z* = 0.005 nm^2^. This resulted in a significant reduction of the work during binding simulations as reported in the lower section of Figure 10 (unbinding profiles in Figure S12). The correct behavior of *S*(*x*) is confirmed by analyzing the evolution of *S*(*x*) over time, as reported in the lower section of Figure 10. *S*(*x*) now evolves linearly over time, similarly to *Ŝ*(*t*), underling that *S*(*x*) closely follows *Ŝ*(*t*). We reconstructed the PMF and estimated the binding free energy for these simulations, resulting in Δ*F*_b_^°^ = −7.8 ± 0.9 kcal/mol. The dissipated work encountered during binding simulations with *Z* = 0.005 nm^2^ is strongly decreased compared to the value obtained with *Z* = 0.05 nm^2^, as reported in Table 5.

As a result, the binding free energy estimate defined with Path 1 and *Z* = 0.005 nm^2^ better aligns with the one defined with Path 2 (which includes the pocket degrees of freedom). Differently, as all trajectories obtained with Path 2 including the pocket were already in the stiff-spring approximation there was no need to tighten the wall. For comparison, the results of all T4 Lysozyme paths obtained using the Jarzynski estimator are reported in Table S4.

Results overall highlight the strong impact of PCVs definition on binding free energies, especially for challenging and complex systems. This underscores the need to include all degrees of freedom relevant to the process, such as protein rearrangements, when defining reference paths for PCVs. Moreover, it is crucial to adapt the value of the PCV *Z* at which the harmonic wall becomes active according to the system size and behavior to ensure the system evolves in the stiff-spring approximation.

By including these enhancements we obtain better results for the T4 Lysozyme system, albeit not in perfect agreement with the experimental affinity. As widely recognized in the literature, systems with buried pockets, such as this one, pose a significant challenge when using geometric collective variable approaches, with alchemical routes being more suitable for buried pockets.^34^ Path-based methods are in general more computationally demanding and require higher human intervention compared to alchemical methods. However, systems with deeply buried pockets can be computationally expensive also for alchemical methods, as buried protein pockets may require long simulation times to be completely solvated after the ligand annihilation. Moreover, path-based methods can provide a higher degree of information than alchemical methods and may be the preferred choice in highly complex systems, for instance in protein–protein binding free energy calculations.^56^ This highlights the continued importance of geometrical routes in biophysical computations.^56^

Through the careful refinements of the pipeline presented here, we pushed the method to its practical limits, successfully achieving modest dissipation protocols, even in this highly complex system.

## 4 Conclusion

When performing nonequilibrium simulations, two major problems arise: optimally sampling the transformation process and optimally reconstructing the free energy difference from these samples. While the second issue has been solved with the Crooks Fluctuation Theorem^8^ (which is optimal in reconstruction efficiency), the first problem remains unsolved. In particular, in this nonequilibrium setting, the target quantity to minimize during sampling is the dissipated work. Hence, the goal is to find optimal transformation routes that lead to minimally dissipative paths.

In this work, we proposed practical guidelines to refine our semiautomated protocol for free energy calculations using path-based nonequilibrium simulations, with a focus on identifying and mitigating the causes of dissipation. Our findings underscore the significant influence of both the water model and the parametrization of the path collective variables on the convergence rate of nonequilibrium estimators. The choice of water model plays a critical role: although water models such as OPC and TIP4P may seem superior in reproducing the properties of pure water, it is essential to consider that the accuracy of thermodynamic simulations of solvated molecules depends on a balance between solvent–solvent, solute–solute, and solute–solvent interactions. As a result, water models optimized for pure water may not always offer the best performance in complex systems and in nonequilibrium scenarios. Interestingly, TIP3P, despite its less accurate representation of pure water, showed better convergence when coupled with AMBER protein force fields in 2-sided nonequilibrium simulations. This can be attributed to the lower viscosity of TIP3P compared to the other water models tested. The reduced viscosity results in a lower heat transfer during nonequilibrium simulations, which enables TIP3P to dissipate significantly less, accelerating the convergence of free energy calculations. These findings are corroborated by two model systems. Furthermore, since water plays both a structural and dynamic role in regulating the binding process through a complex rearrangement of noncovalent bonds, further studies will explore the potential of including water molecules in the path definition. We should also mention that choosing a water model to minimize dissipation is frankly a pragmatic approach: the ideal solution would indeed require the discovery and operative definition of a time-dependent Hamiltonian (a path) that, independently from the water model employed, minimizes heat transfer.

While we did not achieve chemical accuracy in the third test complex (T4 Lysozyme), we successfully optimized the definition of the reference path and collective variables (PCVs) in order to minimize dissipation in nonequilibrium strategies. Importantly, incorporating protein pocket degrees of freedom in the reference path proved largely beneficial. These results underscore the significant challenges associated with buried binding pockets. Interestingly, even MetaDynamics struggled to provide accurate estimates (see Supporting Information), despite not leveraging work for its estimates. Consistently to the literature, systems with buried pockets present substantial difficulties when employing geometric collective variable approaches. Nevertheless, through careful refinement of several aspects of PCVs definition, we pushed the method to its practical limits. In a related study (manuscript under preparation by our group), we found that for systems with a solvent-exposed pocket, the inclusion of pocket atoms in the PCVs definition is sufficient not only to minimize dissipation but also to provide accurate binding free energy estimates.

We hope that our guidelines and recommendations will facilitate the application of nonequilibrium path-based approaches in drug design. Given their inherent parallel nature, these methods hold considerable promise for future research and practical use in the field.

## Supporting Information Available

(1) Additional results for trypsin-benzamidine; (2) Additional results for GSK-3β; (3) Water properties; (4) Additional results for T4 Lysozyme; (5) Well-tempered MetaDynamics study of trypsin-benzamidine and T4 Lysozyme. (PDF)
