# Supplementary material for: Nonequilibrium Binding Free Energy Simulations: Minimizing Dissipation
Source: J Chem Theory Comput. 2025 Feb 5;21(4):2079–94. doi: 10.1021/acs.jctc.4c01453 (PMC11866923; doi:10.1021/acs.jctc.4c01453)
Supplement: Supplementary file 1 — ct4c01453_si_001.pdf [file ct4c01453_si_001.pdf]

Supporting Information for: Non-equilibrium binding free energy simulations: minimizing dissipation

Eleonora Serra,[†,‡,‖] Alessia Ghidini,[¶,‖] Sergio Decherchi,[*,§] and Andrea Cavalli[¶,‡]

†*Department of Pharmacy and Biotechnology (FaBiT), Alma Mater Studiorum - University of Bologna, via Belmeloro 6, 40126 Bologna, Italy*

‡*Computational & Chemical Biology, Fondazione Istituto Italiano di Tecnologia, via Morego 30, 16163 Genoa, Italy*

¶*Centre Européen de Calcul Atomique et Moléculaire (CECAM), Ecole Polytechnique Fédérale de Lausanne, 1015 Lausanne, Switzerland*

§*Data Science and Computation Facility, Fondazione Istituto Italiano di Tecnologia, via Morego 30, 16163 Genoa, Italy*

‖*These authors contributed equally to the work.*

E-mail: sergio.decherchi@iit.it,andrea.cavalli@epfl.ch

Additional results for TRY-BEN

In this section, the work profiles obtained from SMD simulations at different pulling speeds and the corresponding standard binding free energies estimated with the Jarzynski estimator are reported for the TRY-BEN system modeled with different water force fields.

Specifically, the work profiles from the 10 ns simulations are shown in Figure S1 and Figure S2, depicting the unbinding and binding events, respectively.

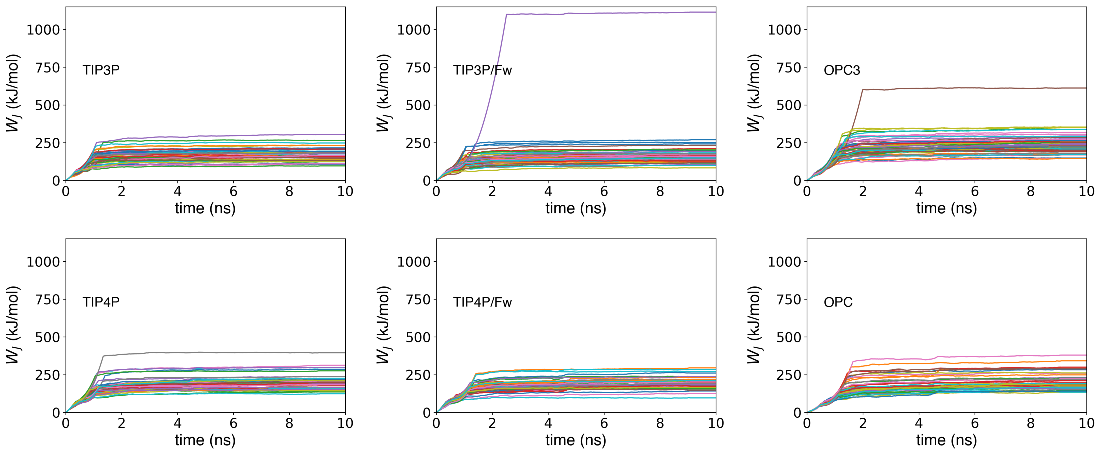

Figure S1: Unbinding work curves for 10 ns simulations of the TRY-BEN system solvated with different water models.

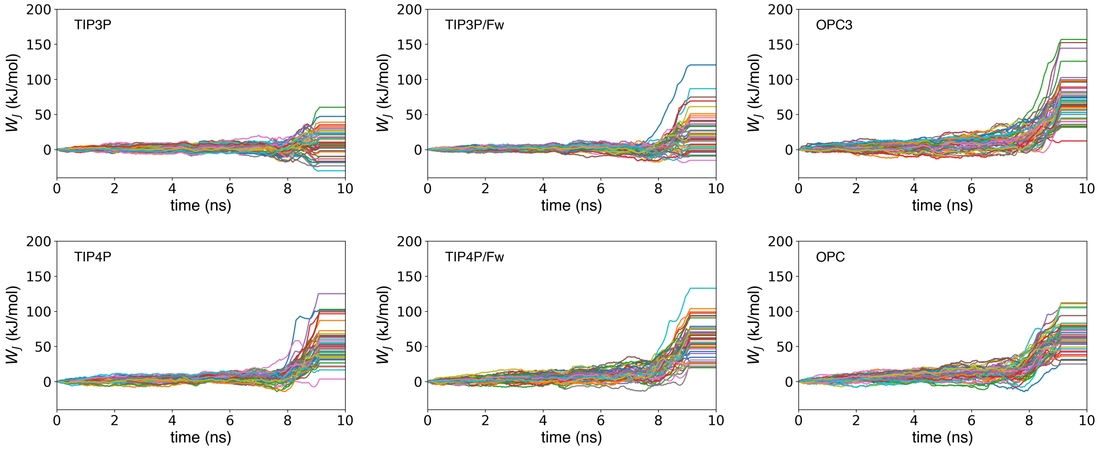

Figure S2: Binding work curves for 10 ns simulations of the TRY-BEN system solvated with different water models.

Additionally, Figure S3 and Figure S4 present the work profiles for the unbinding and binding events at a simulation time of 100 ns, respectively.

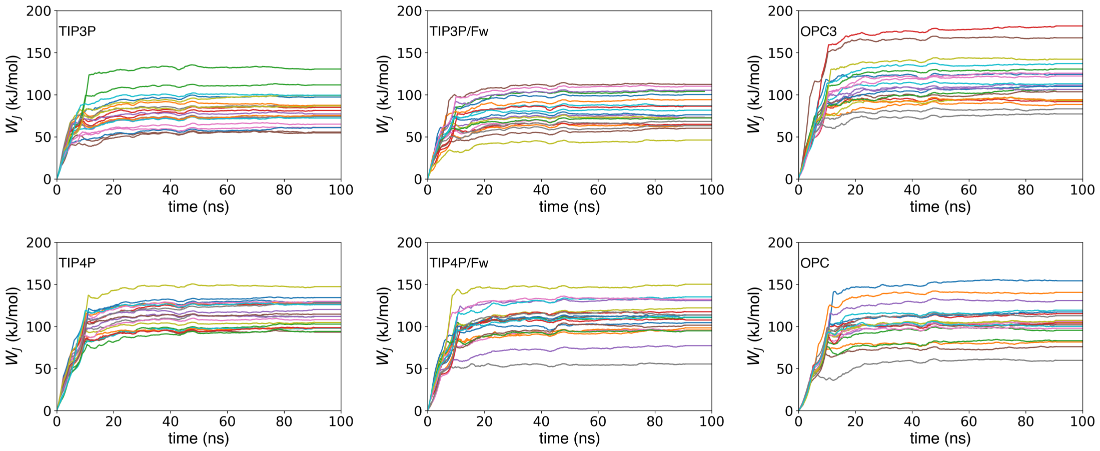

Figure S3: Unbinding work curves for 100 ns simulations of the TRY-BEN system solvated with different water models.

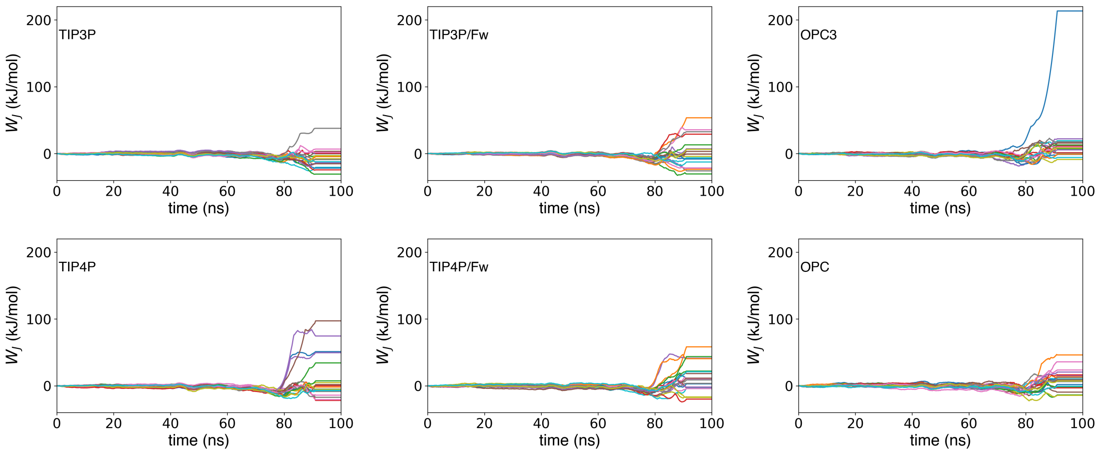

Figure S4: Binding work curves for 100 ns simulations of the TRY-BEN system solvated with different water models.

Finally, Table 1 provides a summary of the standard binding free energies calculated using the Jarzynski estimator for both binding and unbinding simulations across different water models.

Table 1: Standard binding free energies (kcal/mol) computed with the Jarzynski estimator (from unbinding and binding simulations) for TRY-BEN solvated with different water models for different simulation times.

**MODEL**	**10 ns**		**50 ns**		**100 ns**	
	$\Delta\hat{F}_{J,u}$	$\Delta\hat{F}_{J,b}$	$\Delta\hat{F}_{J,u}$	$\Delta\hat{F}_{J,b}$	$\Delta\hat{F}_{J,u}$	$\Delta\hat{F}_{J,b}$
**TIP3P**	-20 ± 1	-4 ± 2	-14.3 ± 0.5	-4 ± 1	-11 ± 1	-4 ± 2
**TIP3P/Fw**	-16 ± 1	-2 ± 1	-14.5 ± 0.8	-4 ± 2	-9 ± 1	-5 ± 1
**OPC3**	-28 ± 2	0 ± 3	-17.7 ± 0.9	-2 ± 2	-15 ± 1	-1 ± 1
**TIP4P**	-25 ± 2	-1 ± 4	-15.8 ± 1	-3 ± 3	-18.6 ± 0.9	-2 ± 2
**TIP4P/Fw**	-23 ± 2	0.1 ± 0.9	-13 ± 2	-2 ± 6	-11.7 ± 0.3	-2 ± 3
**OPC**	-22 ± 1	0 ± 2	-16.9 ± 0.6	-1 ± 1	-10 ± 2	-2 ± 2

As can be seen, the TIP3P model, both in its flexible and rigid versions, leads to $\Delta\hat{F}_{J,u}$ and $\Delta\hat{F}_{J,b}$ results in best agreement with the experimental affinity for TRY-BEN.

Additional results for GSK-3β

In this section, the work profiles obtained from SMD simulations at different pulling speeds and the corresponding standard binding free energies estimated with the Jarzynski estimator are reported for the GSK-3β system modelled with different water force fields.

In particular, the work profiles from the 10 ns SMD are reported in Figure S5 and Figure S6, depicting the unbinding and binding events, respectively.

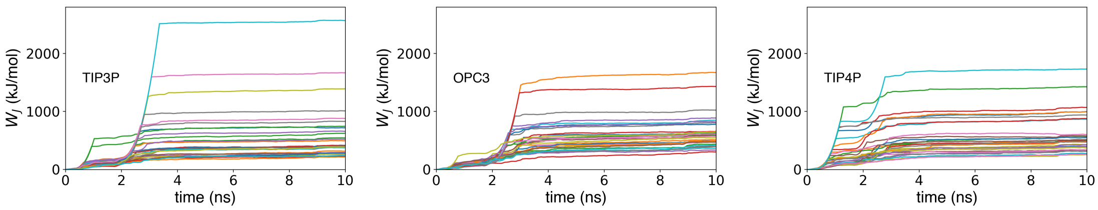

Figure S5: Unbinding work curves for 10 ns simulations of the GSK-3β system solvated with different water models.

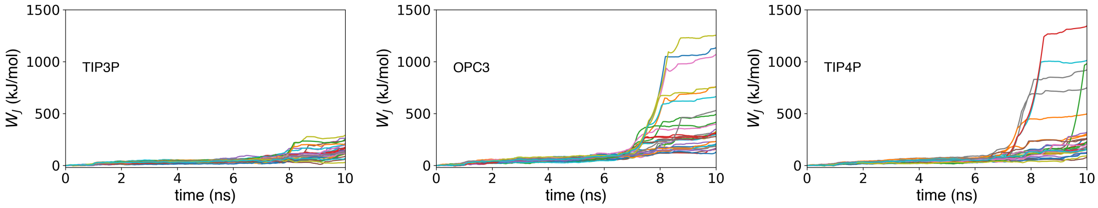

Figure S6: Binding work curves for 10 ns simulations of the GSK-3β system solvated with different water models.

Moreover, Figure S7 and Figure S8 report the work profiles for the unbinding and binding events at a simulation time of 100 ns, respectively.

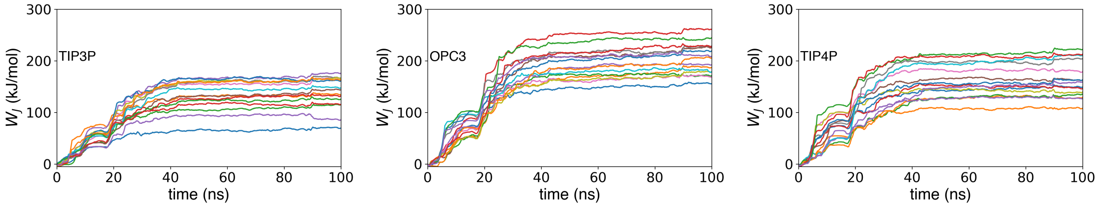

Figure S7: Unbinding work curves for 100 ns simulations of the GSK-3β system solvated with different water models.

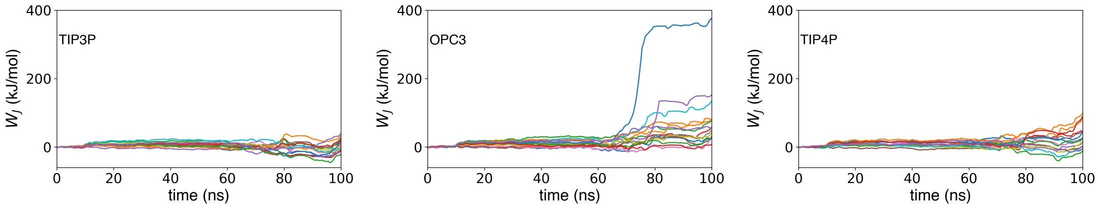

Figure S8: Binding work curves for 100 ns simulations of the GSK-3β system solvated with different water models.

In the end, Table 2 summarizes the standard binding free energies calculated using the Jarzynski estimator for both binding and unbinding simulations using different water models. As can be seen from Table 2, also for GSK-3β, the TIP3P model leads to $\Delta\hat{F}_{J,u}$ and $\Delta\hat{F}_{J,b}$ results in best agreement with the experimental affinity.

Table 2: Standard binding free energies (kcal/mol) computed with the Jarzynski estimator (from unbinding and binding simulations) for GSK-3β solvated with different water models for different simulation times.

**MODEL**	**10 ns**		**50 ns**		**100 ns**	
	$\Delta\hat{F}_{J,u}$	$\Delta\hat{F}_{J,b}$	$\Delta\hat{F}_{J,u}$	$\Delta\hat{F}_{J,b}$	$\Delta\hat{F}_{J,u}$	$\Delta\hat{F}_{J,b}$
**TIP3P**	-41.3 ± 0.8	5 ± 13	-21 ± 1	-3 ± 2	-15.6 ± 0.4	-7 ± 2
**TIP4P**	-42 ± 2	6 ± 10	-28.8 ± 0.9	2 ± 2	-24.7 ± 0.6	-4 ± 4
**OPC3**	-41 ± 2	8 ± 4	-35 ± 3	2 ± 4	-34.3 ± 0.6	-2 ± 3

Water properties

For completeness, Table 3 presents the experimental values of key water properties alongside the values calculated using the water models discussed in this study.

Table 3: Experimental values of main water properties compared to values calculated with water models discussed here.

**MODEL**	**Density (g/cm^3)**	**Coord. No.**	**Surface tension (mN/m)**	**Dielectric constant**	**Self-diffusion coeff. (10^{-5} cm^2/s)**
**TIP3P**	0.980 ± 0.006	6.239 ± 0.001	47.0 ± 0.2	95 ± 3	5.72 ± 0.04
**TIP3P/Fw**	1.027 ± 0.006	5.3 ± 0.3	55.2 ± 0.4	197 ± 2	3.8 ± 0.1
**OPC3**	0.991 ± 0.006	4.9 ± 0.2	61.0 ± 0.2	79 ± 1	2.28 ± 0.02
**TIP4P**	0.994 ± 0.006	5.14 ± 0.001	52.2 ± 0.2	51.3 ± 0.5	2.57 ± 0.04
**OPC**	0.997 ± 0.005	5.1971 ± 0.0005	70.1 ± 0.2	78 ± 1	2.27 ± 0.02
**Expt.**	0.997	4.7	71.99	78.3	2.30

Additional results for T4 Lysozyme

This section presents additional results for the T4 Lysozyme system. Specifically, the work profiles from 100 ns SMD simulations using PCVs and various reference paths are shown in Figure S9, Figure S10, Figure S11, and Figure S12. In these plots, the unbinding work curves are displayed on the left side, while the binding work curves are shown on the right side.

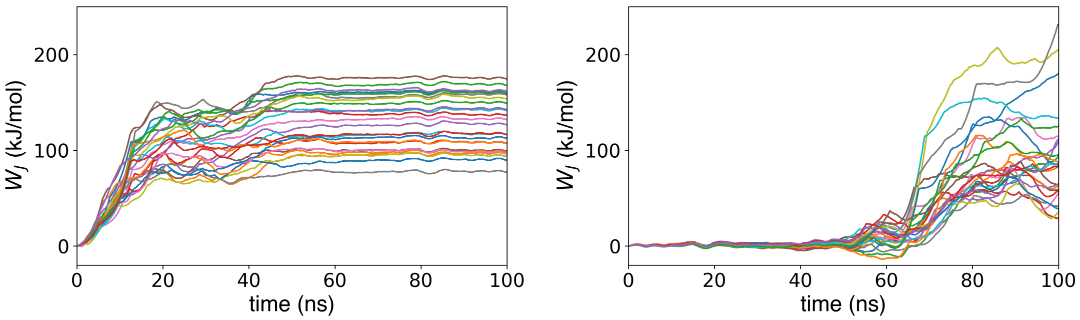

Figure S9: Unbinding (left) and binding (right) work curves of 100 ns for the T4 Lysozyme system with Path 1 defined by using ligand atoms only.

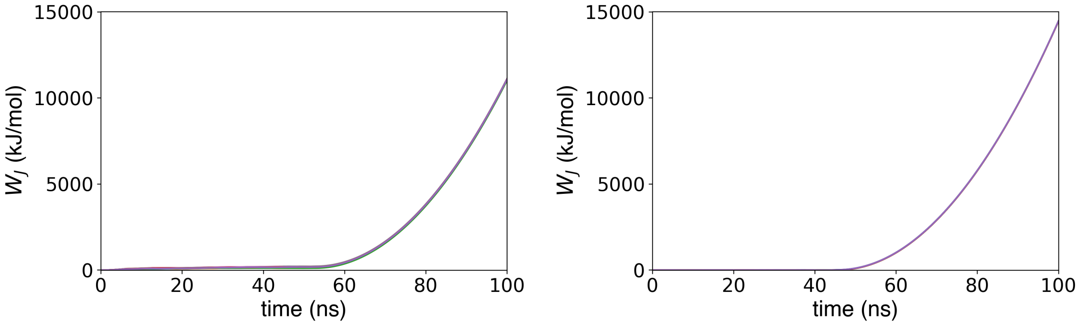

Figure S10: Unbinding (left) and binding (right) work curves of 100 ns for the T4 Lysozyme system with Path 2 defined by using ligand atoms only.

Additionally, Figure S13 and Figure S14 depict the unbinding (left) and binding (right) work curves from 100 ns SMD simulations, with the harmonic wall centered at $Z = 0.005$ nm^2 for Path 1 and Path 2, respectively. Moreover, Figure shows the evolution of $S(x)$

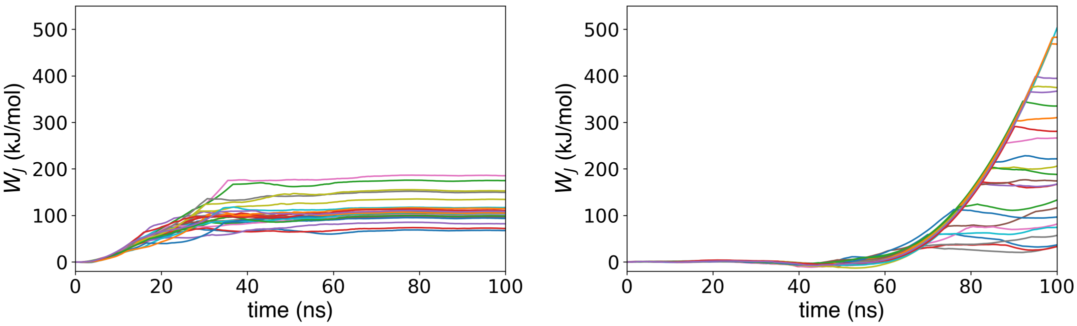

Figure S11: Unbinding (left) and binding (right) work curves of 100 ns for the T4 Lysozyme system with Path 1 defined with ligand and pocket atoms.

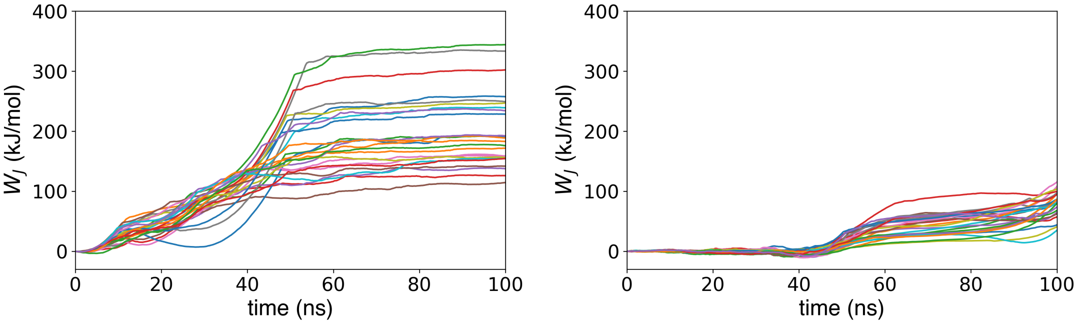

Figure S12: Unbinding (left) and binding (right) work curves of 100 ns for the T4 Lysozyme system with Path 2 defined with ligand and pocket atoms.

during binding simulation of 2RBN using Path 2 with ligand and pocket atoms centering the harmonic wall in $Z = 0.05$ nm^2 (upper section) and $Z = 0.005$ nm^2 (lower section).

Table 4 reports standard binding free energies (kcal/mol) estimated using the Jarzynski and CFT estimators for 100 ns SMD simulations using different paths. Results of Path 2 are not reported because the high dissipated work prevented their calculation.

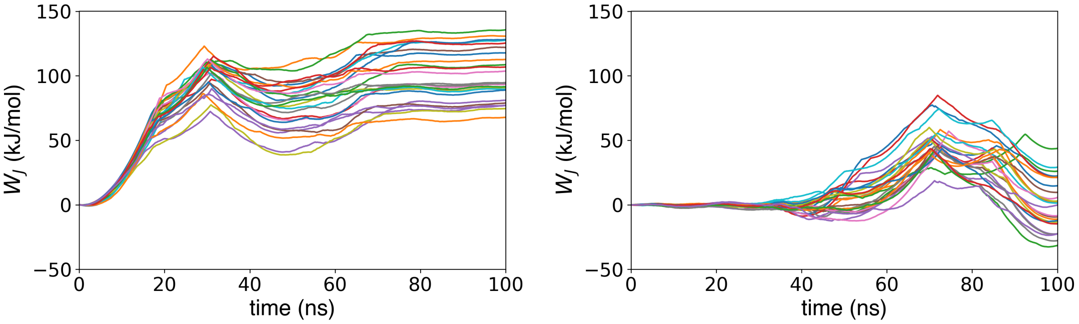

Figure S13: Unbinding (left) and binding (right) work curves of 100 ns SMD simulations with the harmonic wall centered in $Z = 0.005$ nm^2 for the T4 Lysozyme system with Path 1 defined with ligand and pocket atoms.

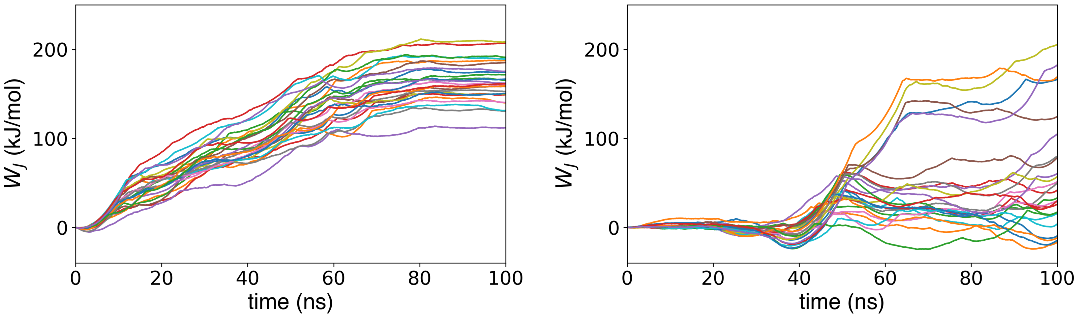

Figure S14: Unbinding (left) and binding (right) work curves of 100 ns SMD simulations with the harmonic wall centered in $Z = 0.005$ nm^2 for the T4 Lysozyme system with Path 2 defined with ligand and pocket atoms.

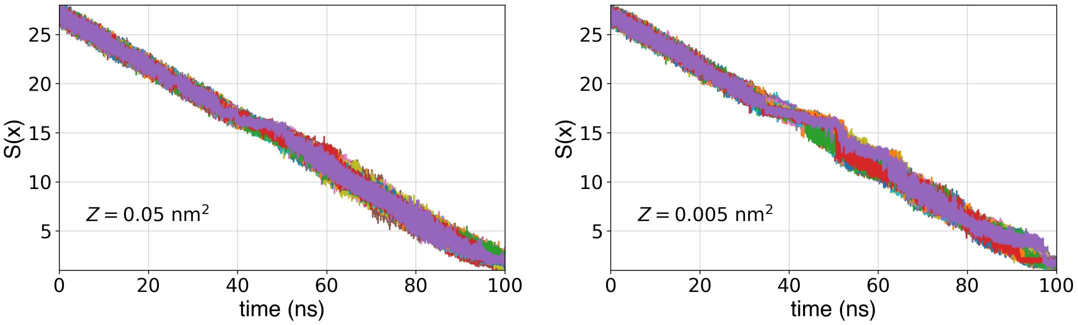

Figure S15: Evolution of $S(x)$ during binding simulation of 2RBN using Path 2 with ligand and pocket atoms centering the harmonic wall in $Z = 0.05$ nm^2 (upper section) and $Z = 0.005$ nm^2 (lower section).

Table 4: Standard binding free energies (kcal/mol) estimated using the Jarzynski and CFT estimators for 100 ns SMD simulations of the T4 Lysozyme using different paths. Values for Path 2 are missing as the high dissipated work prevented their calculation.

**Path**	**JE binding**	**JE unbinding**	**CFT**
**Path 1**	-1 ± 10	-22±0.8	-10±3
**Path 2**	*	*	*
**Path 1 with Pocket**	1 ± 12	-15±1	-2±4
**Path 2 with Pocket**	2±7	-20±4	-8±2
**Path 1 with Pocket and smaller Z**	-4±1	-10±1	-7.8±0.9

Well-Tempered MetaDynamics

To fully validate our results we run also Well-Tempered MetaDynamics simulations. The comparison with Well-Tempered MetaDynamics is rather interesting as this is still a non-equilibrium method (i.e. it employs a time-varying Hamiltonian), yet it does not depends on work values. Conversely, the non-equilibrium approaches based on Crooks/Jarzynski estimators depend on this key quantity.

Well-Tempered MetaDynamics of TRY-BEN

The proper parameter choice for Well-Tempered MetaDynamics simulations varies among the different water models. Specifically, water models associated with a higher viscosity, such as TIP4P, necessitate a higher bias potential in MetaDynamics simulations. Furthermore, one can expect a less prominent impact of dissipation in Well-Tempered MetaDynamics simulations compared to SMD ones, which are more sensitive to kinetics.

After fine-tuning the Well-Tempered MetaDynamics parameters, convergence was achieved at 1.5 μs for all simulations and PMFs were successfully reconstructed. PMFs obtained from Well-Tempered MetaDynamics simulations are reported in Figure S16, while Table 5 summarizes the results.

Table 5: Well-Tempered MetaDynamics parameters for different water models and corresponding binding free energies (kcal/mol).

MODEL	TIP3P	OPC3	TIP4P	OPC
**Height**	0.2	0.5	0.5	0.5
**Bias Factor**	10	15	15	15
**Peace**	750	500	500	500
**Binding Free Energy**	-4.108	-6.966	-6.853	-6.691

Starting from the simplest water model, i.e. TIP3P, results are in contrast with respect to results obtained using our non-equilibrium protocol. In fact, the free energy value computed from Well-Tempered MetaDynamics is less consistent with the experimental value. This

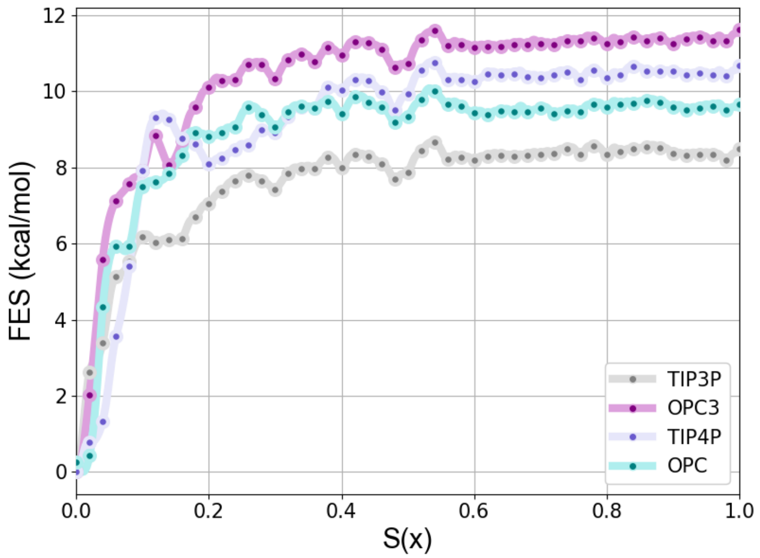

Figure S16: PMFs from Well-Tempered MetaD simulations with different water models.

finding aligns with our previous hypothesis: while the TIP3P water model may not be the optimal choice overall, it appears to be the most suitable in the context of non-equilibrium SMD simulations.

Despite OPC3 being a 3-point water model, it is evident that we cannot apply the same simulation parameters used for TIP3P. OPC3 characteristics are more similar to water models with higher viscosity, such as TIP4P and OPC. Although the results obtained using our non-equilibrium protocol were similar for TIP3P and OPC3 water models, here we observed the superiority of the OPC3 water model, as emphasized by AMBER developers and in the literature. Indeed, employing Well-Tempered MetaDynamics simulations with OPC3 resulted in a closer alignment with the experimental affinity.

Consistently with our previous observations, while TIP4P may have displayed shortcomings in Steered MD simulations, its superiority over TIP3P in Well-Tempered MetaDynamics simulations is notable. Although TIP4P is not always the most suitable choice with non-equilibrium estimators, in less kinetic-sensitive approaches, such as Well-Tempered MetaDynamics, higher-level water models may indeed offer the best solution. Moreover, also OPC in Well-Tempered MetaDynamics simulation resulted in a closer agreement of the binding

free energy value with the experimental result.

Finally, for completeness, the PMF obtained from Well-Tempered MetaDynamics using the TIP3P water model, with the same simulation parameters applied to other water models, is also presented in Figure 17.

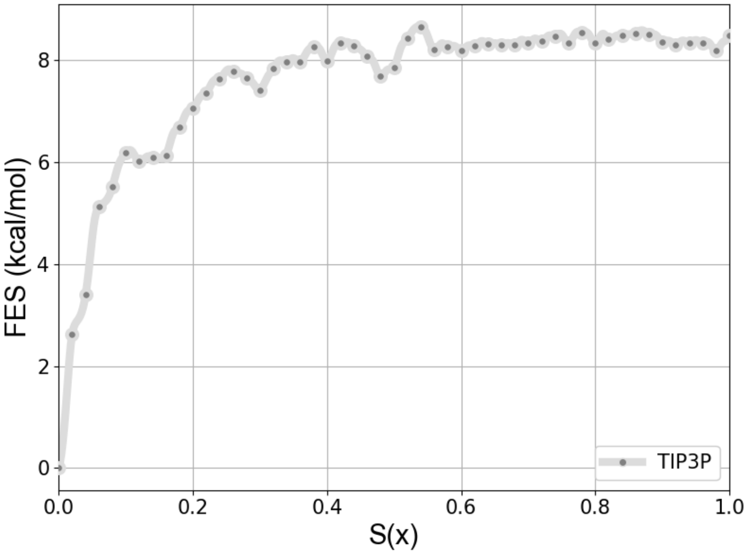

Figure S17: PMF from Well-Tempered MetaD in TIP3P with the same simulation parameters used for the other water models.

Well-Tempered MetaDynamics of T4 Lysozyme

Here we report the Well-Tempered MetaDynamics results for T4 Lysozyme with PCVs and different reference paths. Convergence was achieved at 1 μs for all simulations and PMFs were successfully reconstructed.

According to PMFs (Figures S18), the inaccuracies in binding free energy results obtained from SMD simulations and non-equilibrium estimators are also consistent with those from Well-Tempered MetaDynamics analysis. These findings confirm that this issue is not related to our non-equilibrium protocol but is more likely tied to the correct definition of PCVs and the reference path. Moreover, Figure S19 shows that the definition with Path 2 with only ligand atoms results in a behavior similar to the one observed in SMD simulations. The system remains trapped on the protein surface when $S(x)$ is approximately 39 and 40. This

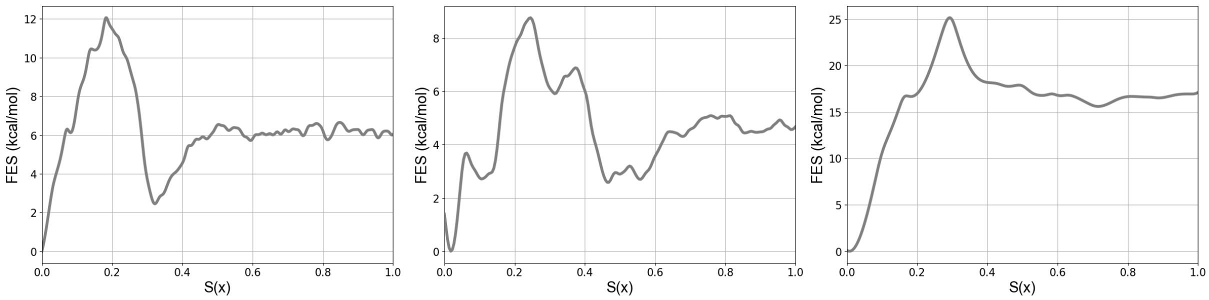

Figure S18: PMFs from Well-Tempered MetaD: a) With the harmonic restraint centered in Z=0.05 nm^2 for the T4 Lysozyme system with Path 1 defined with ligand atoms only. b) With the harmonic restraint centered in Z=0.05 nm^2 for the T4 Lysozyme system with Path 1 defined with ligand and pocket atoms. c) With the harmonic restraint centered in Z=0.05 nm^2 for the T4 Lysozyme system, using Path 2 defined exclusively with ligand atoms.

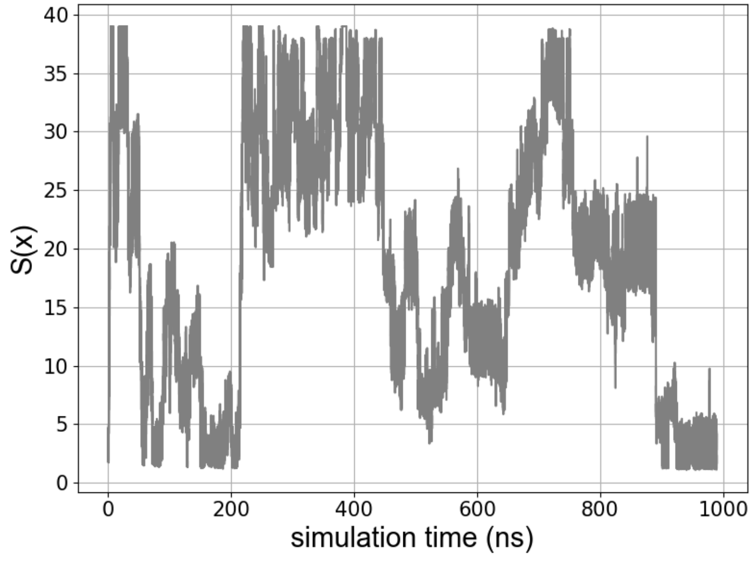

Figure S19: S(X) as a function of the simulation time during Well-Tempered MetaD with the harmonic restraint centered in Z=0.05 nm^2 for the T4 Lysozyme system, using Path 2 defined exclusively with ligand atoms.

underlines the critical role of also incorporating pocket atoms to accurately capture the dynamics of the system. Overall, the Well-Tempered MetaDynamics findings demonstrate that we successfully optimized both the reference path and the collective variables (PCVs) definition. Finally, Well-Tempered MetaDynamics analysis highlight the significant challenges associated with buried binding pockets encountered also in the non-equilibrium SMD case.
